# Supplementary material for: Gfi1b regulates the level of Wnt/β-catenin signaling in hematopoietic stem cells and megakaryocytes
Source: Nat Commun. 2019 Mar 20;10:1270. doi: 10.1038/s41467-019-09273-z (PMC6426870; doi:10.1038/s41467-019-09273-z)
Supplement: Supplementary file 1 — Supplementary Information [file 41467_2019_9273_MOESM1_ESM.pdf]

Gfi1b regulates the level of Wnt/β-catenin signaling in hematopoietic stem cells and megakaryocytes

Shooshtarizadeh et al

Supplementary Figure.1

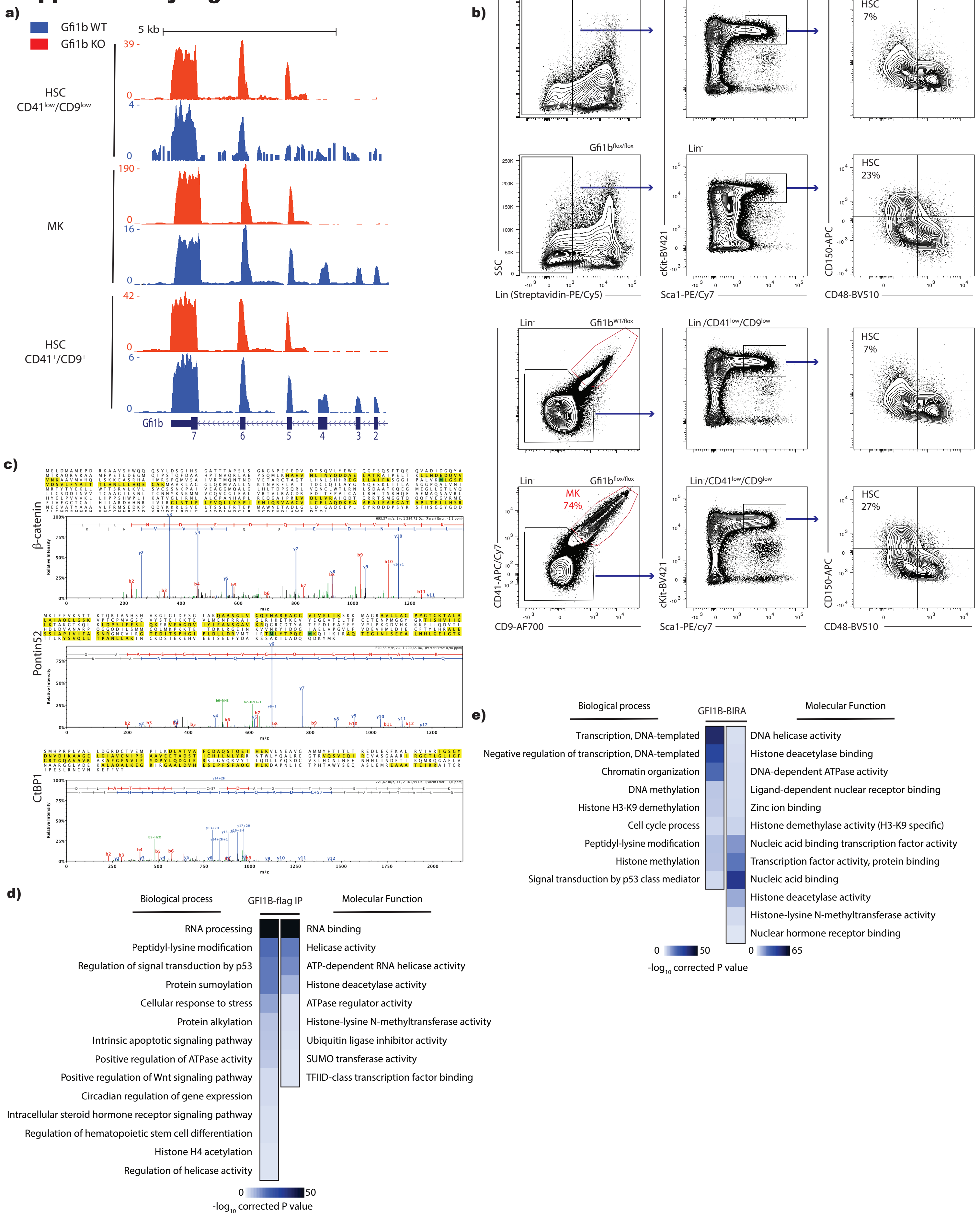

Supplementary Figure 1: Loss of Gfi1b in MKs and HCSs causes their expansion (a) Representative RNA-seq of Gfi1b gene in sorted MKs, HSC and HSC CD41low/CD9low cells showing deletion of exon 2 to 4 following Tamoxifen injection. (b) Representative FACS gating strategy, showing expansion of BM derived MKs and HSCs following deletion of Gfi1b. (c) Peptide sequence of β-catenin, Pontin52 and CtBP1. Peptides identified by mass spectrometry are highlighted and corresponding spectra are shown below. (d) Heat map illustrating biological processes and molecular functions of associated GO terms associated with the proteins found in HEK293 cells expressing GFI1B-Flag after immune-precipiation and mass spectrometric analysis. The enrichment score of each GO term is shown as the -log10 of corrected P values, indicated by different color intensities. (e) Heat map illustrating biological processes and molecular functions of associated GO terms in Flp-In T-REx HEK293 cells expressing GFI1B-BirA*-Flag. The enrichment score of each GO term is shown as the -log10 of corrected P values, indicated by different color intensities.

Supplementary Figure.2

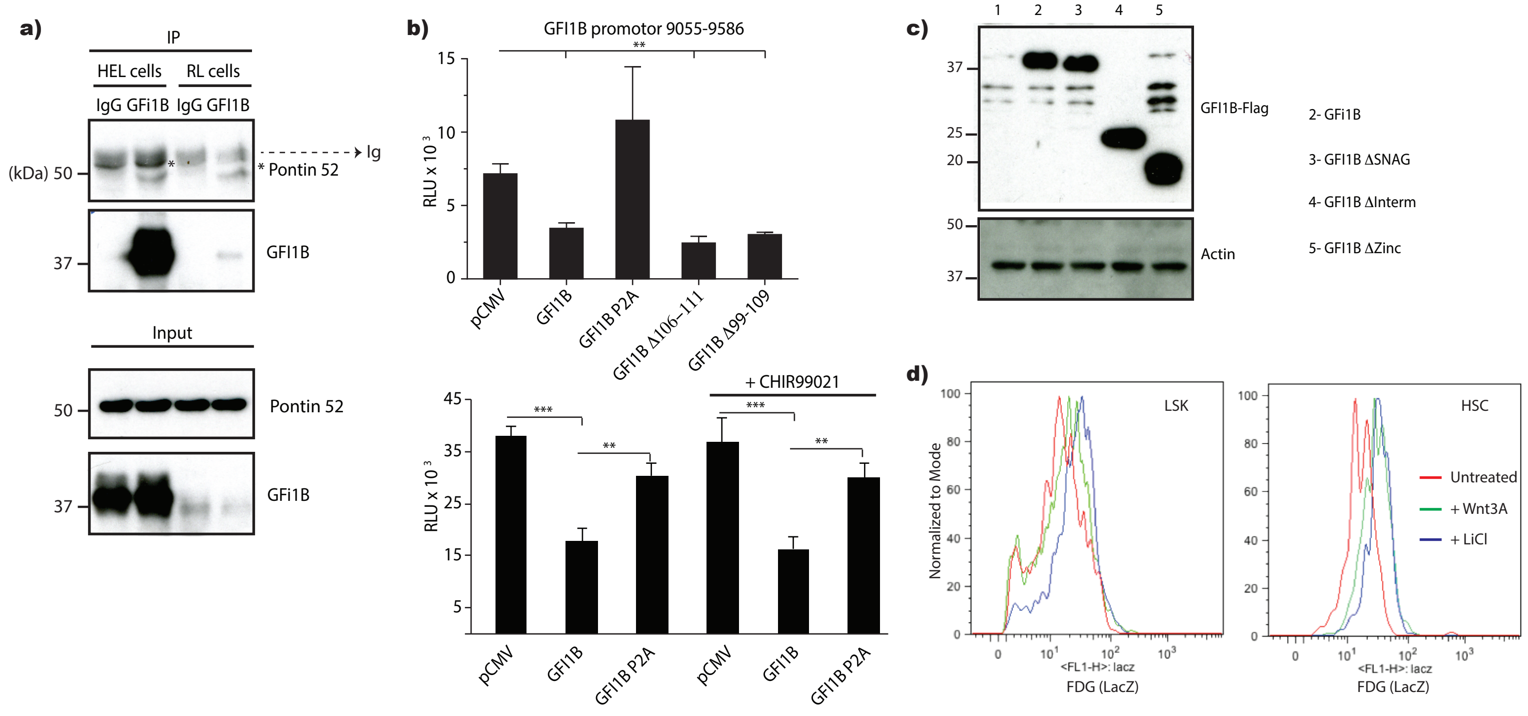

Supplementary Figure 2: GFI1B regulates transcription of a TCF dependent promoter/reporter system (a) Endogenous GFI1B IP shows interaction between GFI1B and Pontin52 in HEL cells. (b) GFI1B promoter luciferase assay in 293T cells. Cells were transfected with indicated vectors to show their ability to inhibit the expression of GFI1B promoter reporter. GFI1B binds to its promoter and inhibits its expression in a SNAG-domain dependent (see GFI1B P2A mutation). The newly identified WRD is not required for the repressor activity of GFI1B (top). GFI1B maintains its repressor activity following activation of Wnt/ β-catenin signaling by treatment with CHIR99021 (bottom) (* $P<0.05$, ** $P<0.001$, *** $P<0.0001$ on a Welch corrected T-test, error bars show s.d). (c) Western blotting on lysates from reporter assay samples in Fig. 3f to check the expression of the transfected vectors. (d) lacZ staining of LSKs and HSCs from Axin II +/lacz mice, following treatment with LiCl and Wnt3a that shows an increase in lacZ MFI.

Supplementary Figure.3

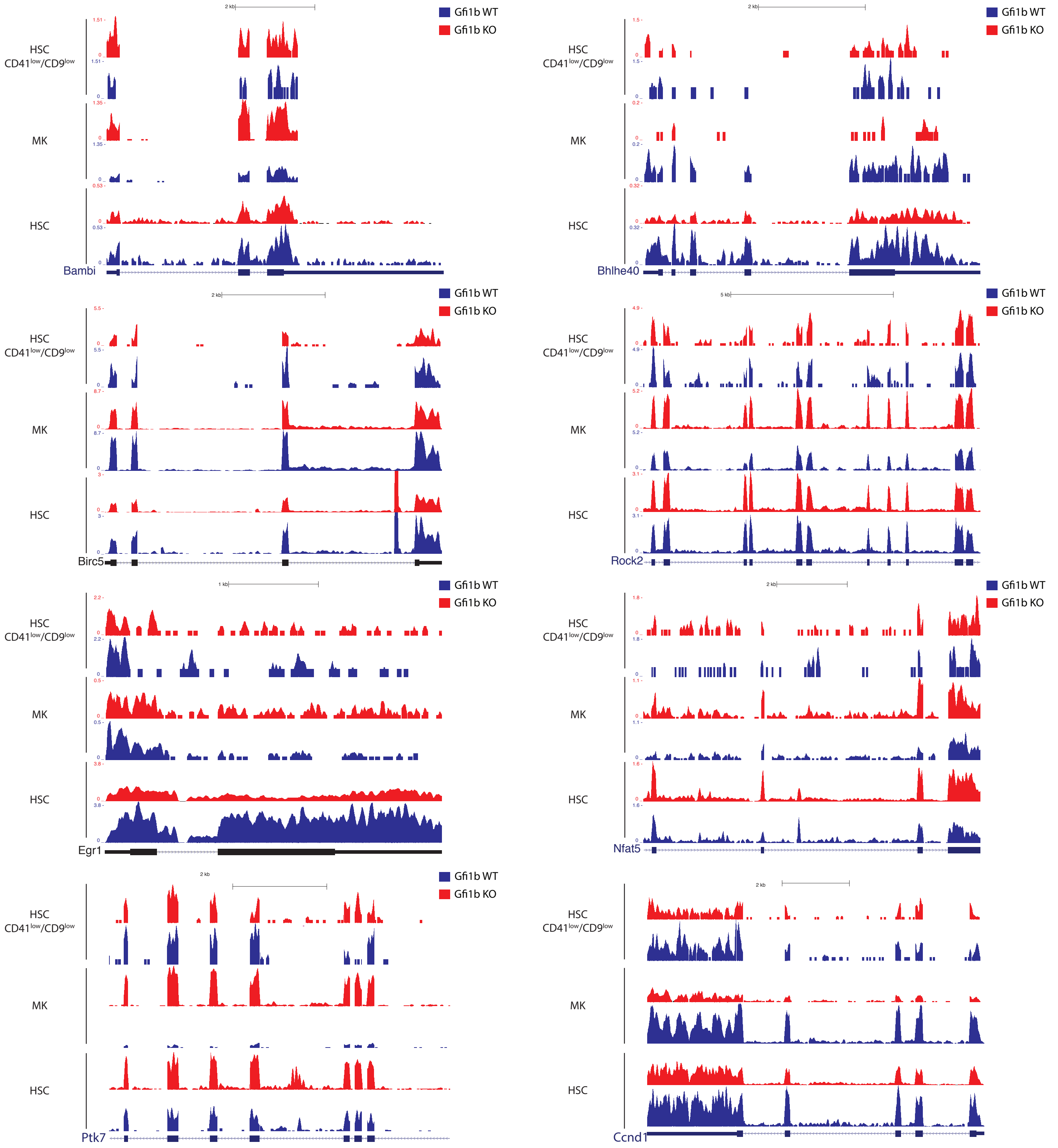

Supplementary Figure 3: Representative RNA-seq data from sorted MKs, HSCs and HSCs CD41low/CD9low Gfi1b WT/KO cells for selected genes.

Supplementary Figure.4

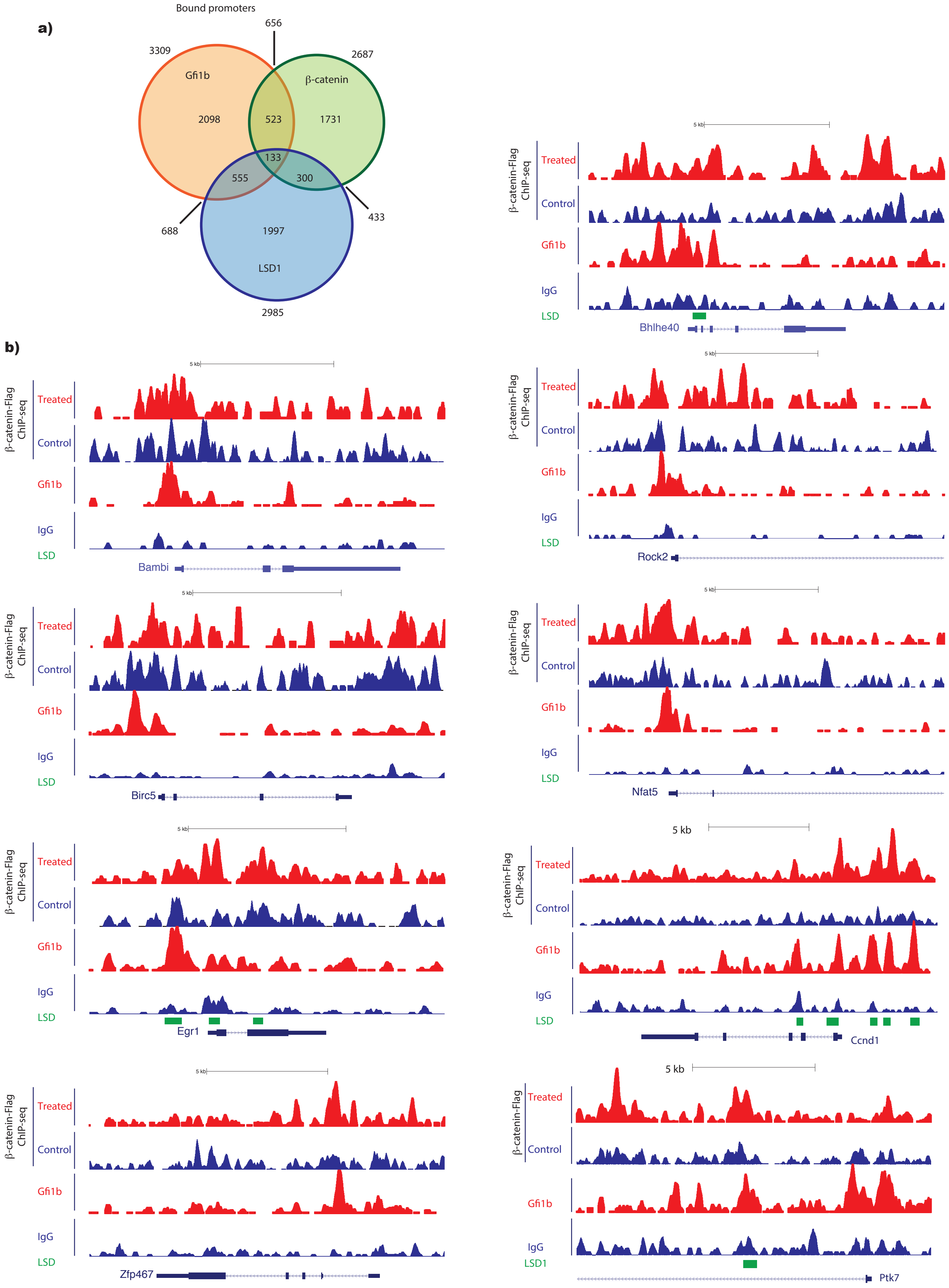

Supplementary Figure 4: Gfi1b, β-catenin and LSD1 occupy common genes promoter targets. Previously published ChIP-seq data for Gfi1b in HPC7 cell line 38, β-catenin and LSD1 in ES cells 39, 40 were used for this meta-analysis. (a) Venn diagram showing gene promoters bound, uniquely and in common, by Gfi1b, β-catenin and LSD1. (b) A representative example for ChIP-seq peaks showing read density at the promoter of selected genes. Note that LSD1 ChIP-seq data is shown as horizontal green bars indicating LSD1 binding.

Supplementary Figure.5

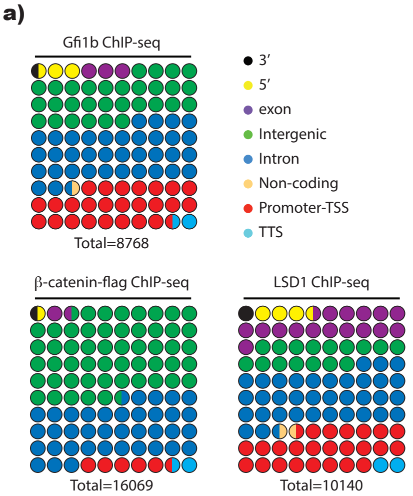

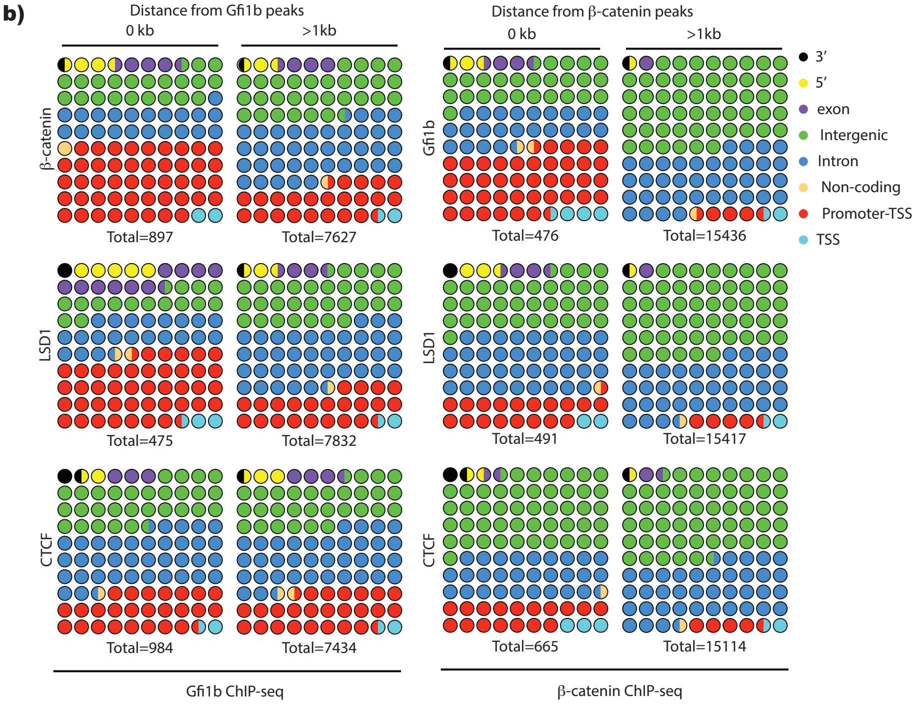

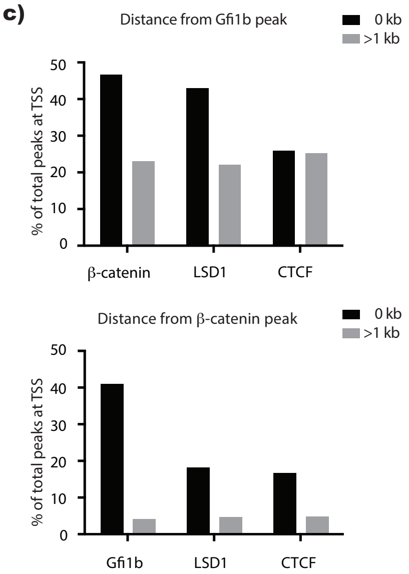

Supplementary Figure 5: Gfi1b and β-catenin occupy common positions at their target genes. (a) Overall distribution of called peaks in each individual previously published ChIP-seq data. (b) Meta-analysis of indicated ChIP-seq data for peak positioning based on their distance to Gfi1b peaks in the used Gfi1b ChIP-seq data set (left panel) or β-catenin peaks in the used β-catenin ChIP-seq dataset (right panel). (c) Frequency of the indicated ChIP-seq peaks at the transcription start site (TSS) based on their distance to Gfi1b peaks in Gfi1b ChIP-seq (top graph) or β-catenin peaks in β-catenin ChIP-seq (bottom graph).

Supplementary Figure.6

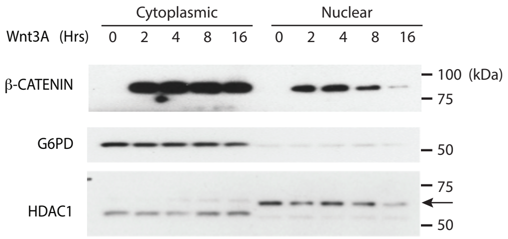

Supplementary Figure 6: Western blot on K562 cells treated with Wnt3A

Supplementary Figure.7

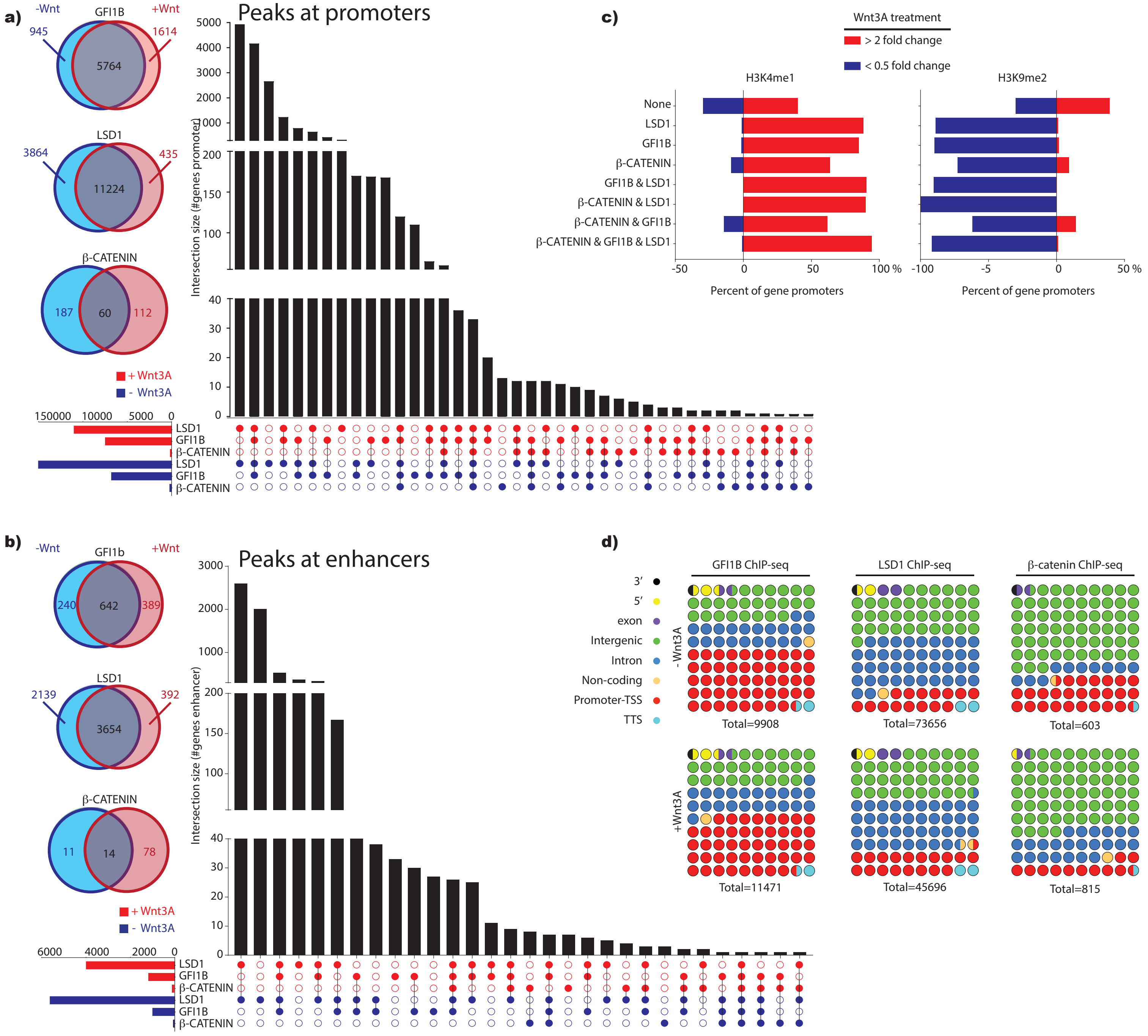

Supplementary Figure 7: GFi1b, β-catenin and LSD1 co-occupy sites at enhancer regions and gene promoters (a-b) Upset plot and Venn diagrams showing number of promoters (in a) and enhancer regions (in b) co-occupied by GFI1B, β-catenin and LSD1 in K562 ChIP-seqs. Venn diagrams show numbers of bound promoters (in a) and enhancers (in b) by each factor individually before and after Wnt3A treatment. (c) Proportion of genes with H3K4me1 and H3K9me2 fold changes at genes promoters targeted as indicated in K562 following treatment with Wnt3A. (d) Overall distribution of called peaks in each individual ChIP-seq data.

Supplementary Figure.8

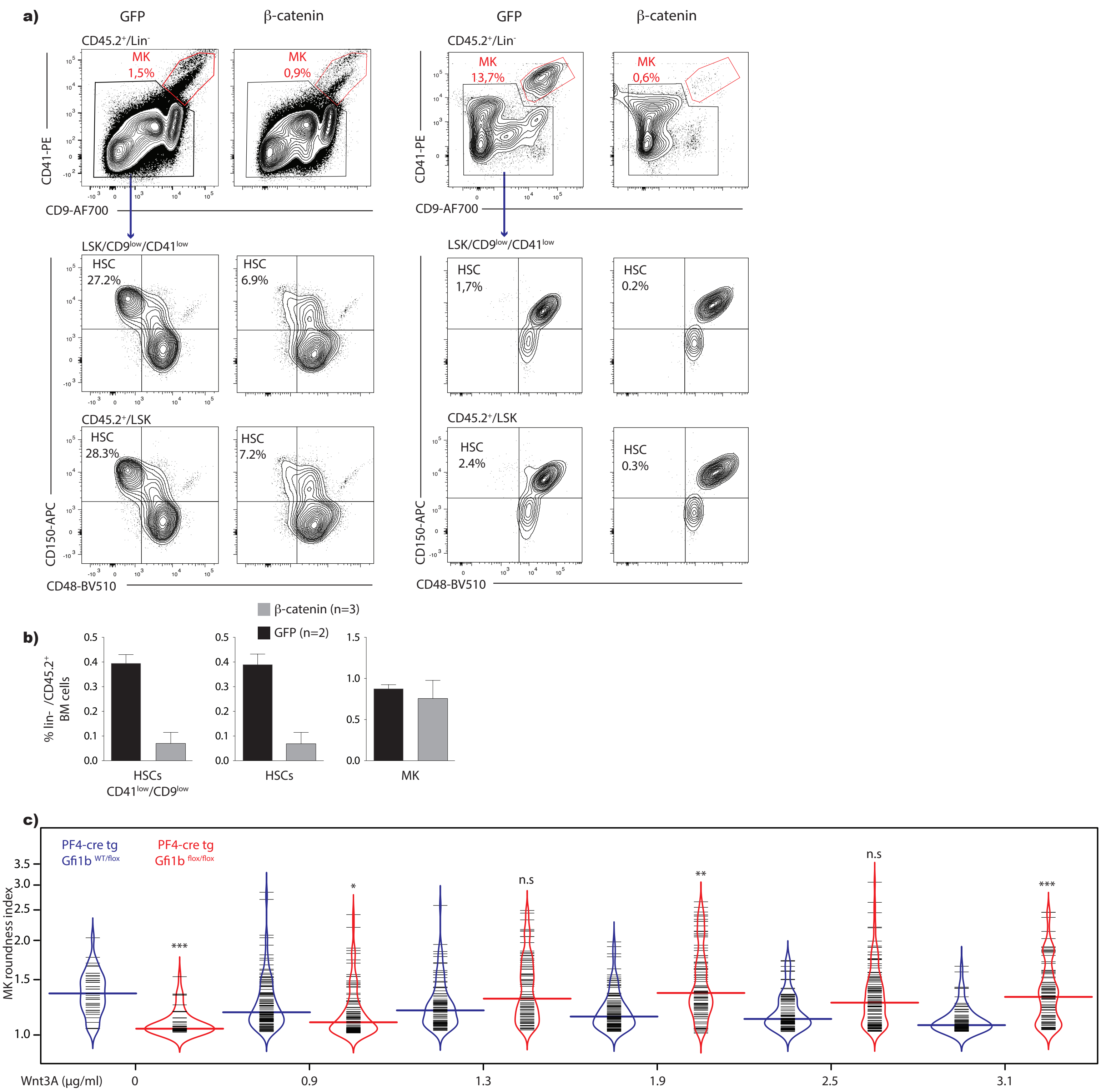

Supplementary Figure 8: Up-regulation of canonical Wnt signaling rescues Gfi1b deficient phenotypes in HSCs and MKs (a-b) Activating the canonical Wnt signaling by retroviral expression of active β-catenin, inhibits HSCs and MKs expansion in Gfi1b KO cells. CD45.2 Gfi1b KO lineage depleted cells were infected with retrovirus expressing active form of β-catenin or GFP. Irradiated CD45.1 mice were transplanted with the infected cells and analyzed by FACS3 4 months post transplantation and HSCs and MKs numbers were quantified. (c) Spreading of Gfi1b WT/KO MKs on Fibronectin coated matrices is affected by Wnt3A treatment at increasing concentrations. Statistical analysis was done for each pair of Gfi1b WT/KO at the given Wnt3A concentration (* $p<0.05$, ** $p<0.001$, *** $p<0.0001$ on a Welch corrected T-test, error bars show s.d)

Fig 2d

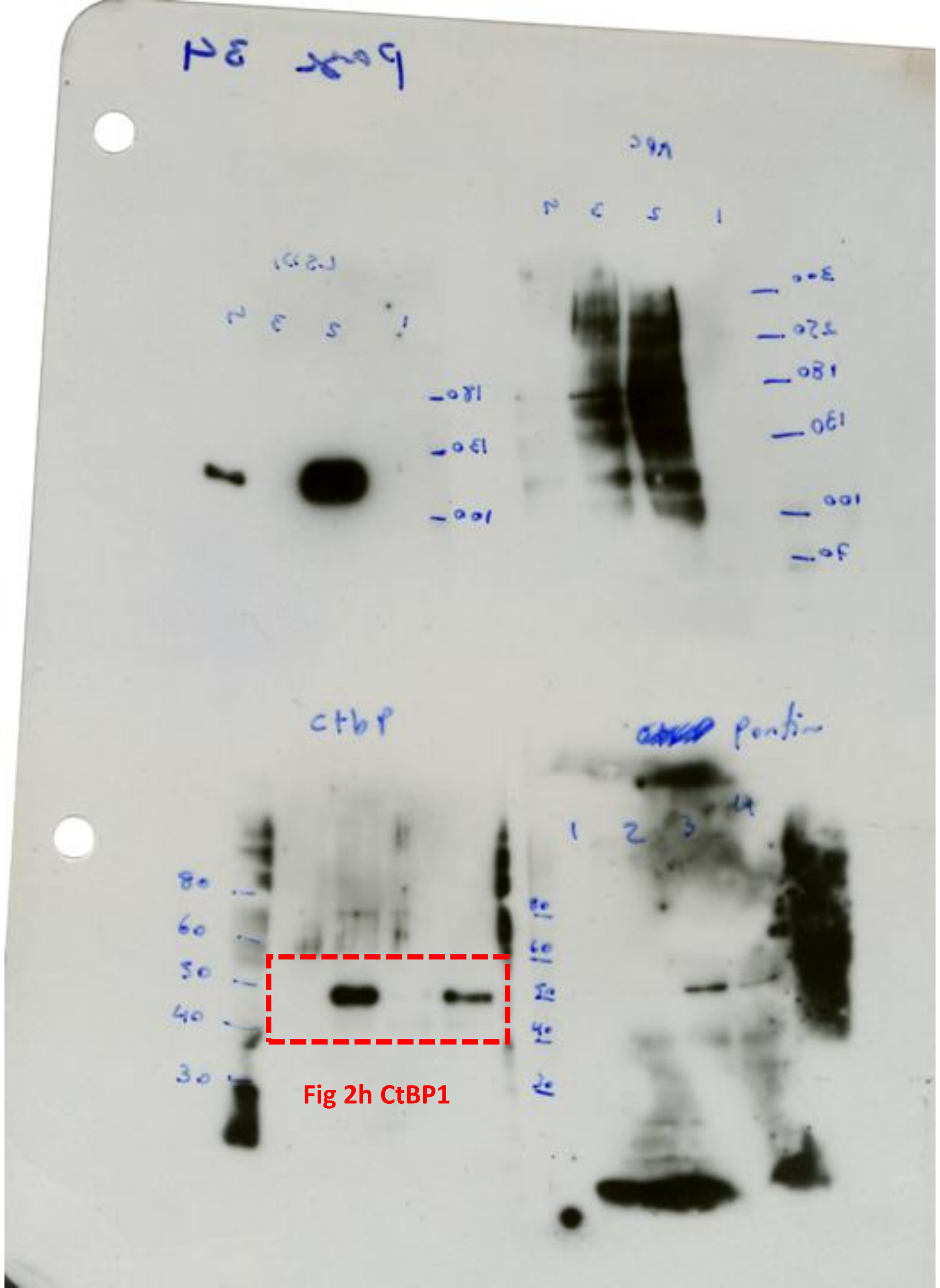
ctbp
Fig 2h CtBP1

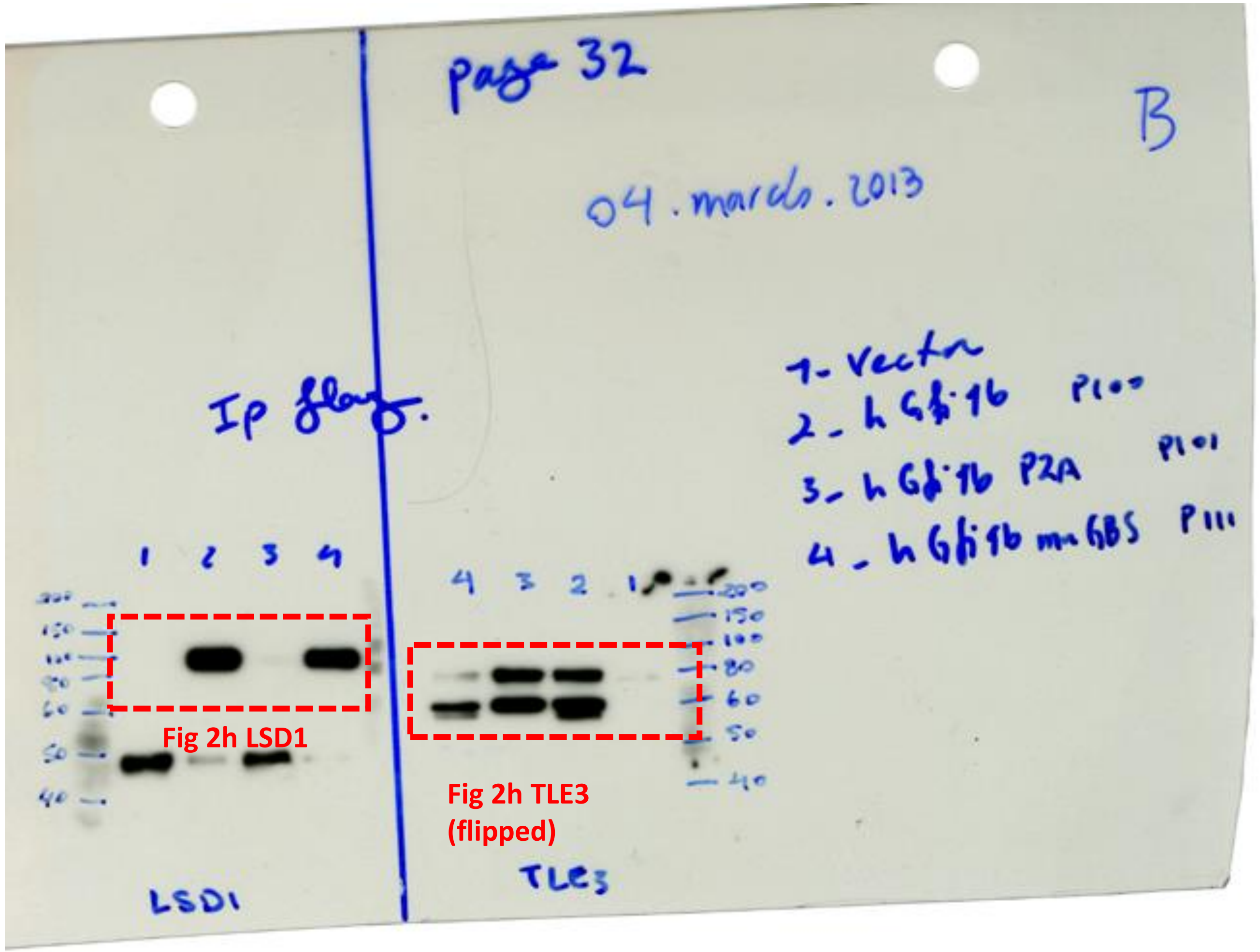
page 32
B
04. march. 2013
Ip flag.
1- vector
2- hGfi1b
3- hGfi1b P2A
4- hGfi1b
1 2 3 4
4 3 2 1
Fig 2h LSD1
Fig 2h TLE3
(flipped)
LSD1
TLE3

Fig 2d

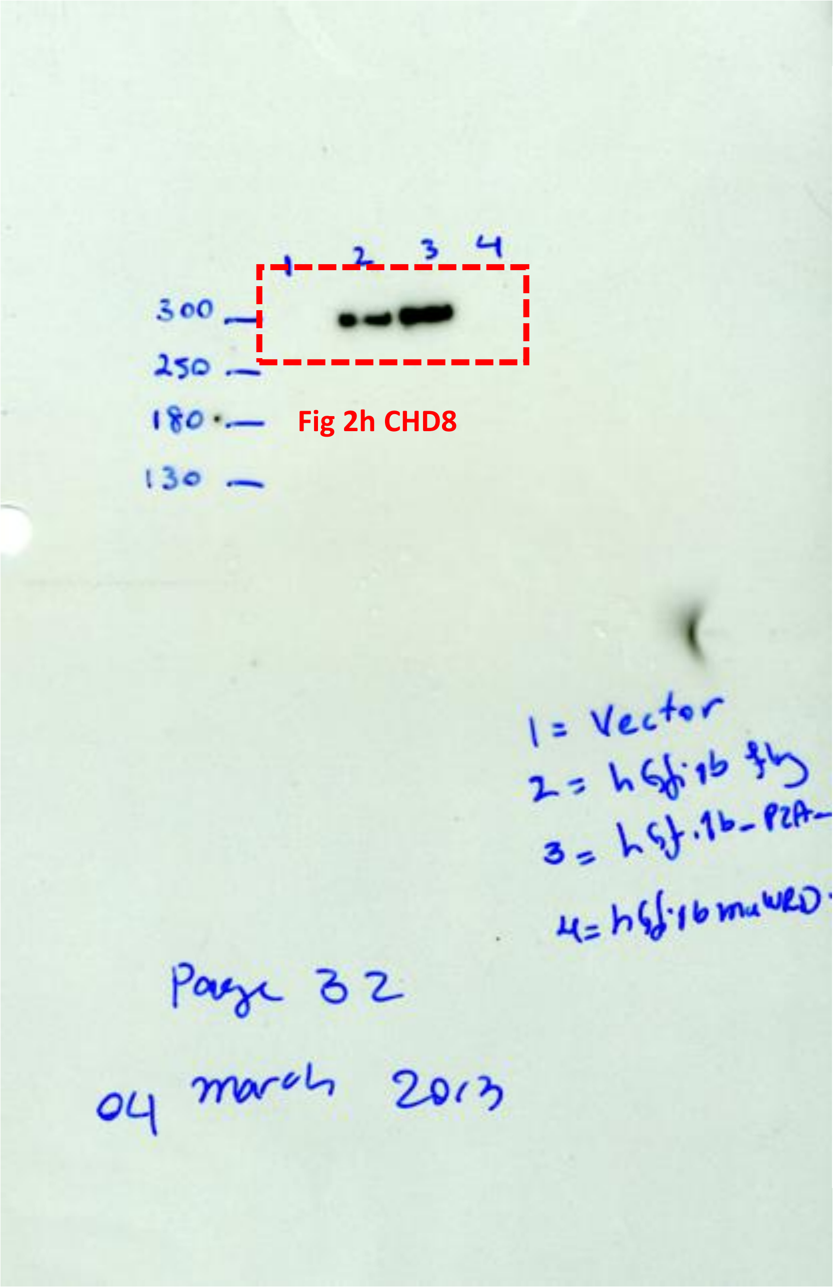
1 2 3 4
300
250
180
130
Fig 2h CHD8
1= Vector
Page 32
04 march 2013

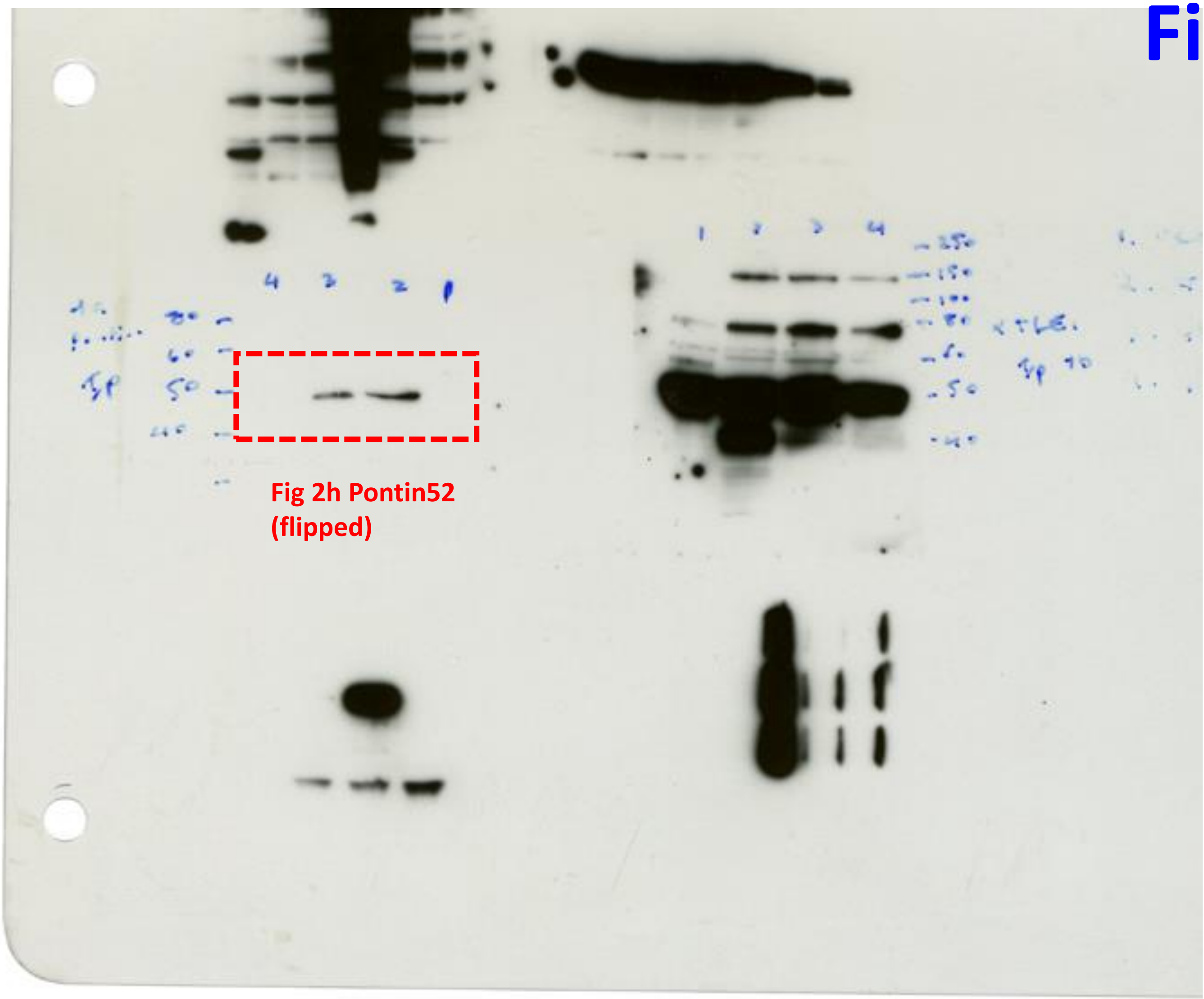
Fig 2h Pontin52
(flipped)

Fig 2d

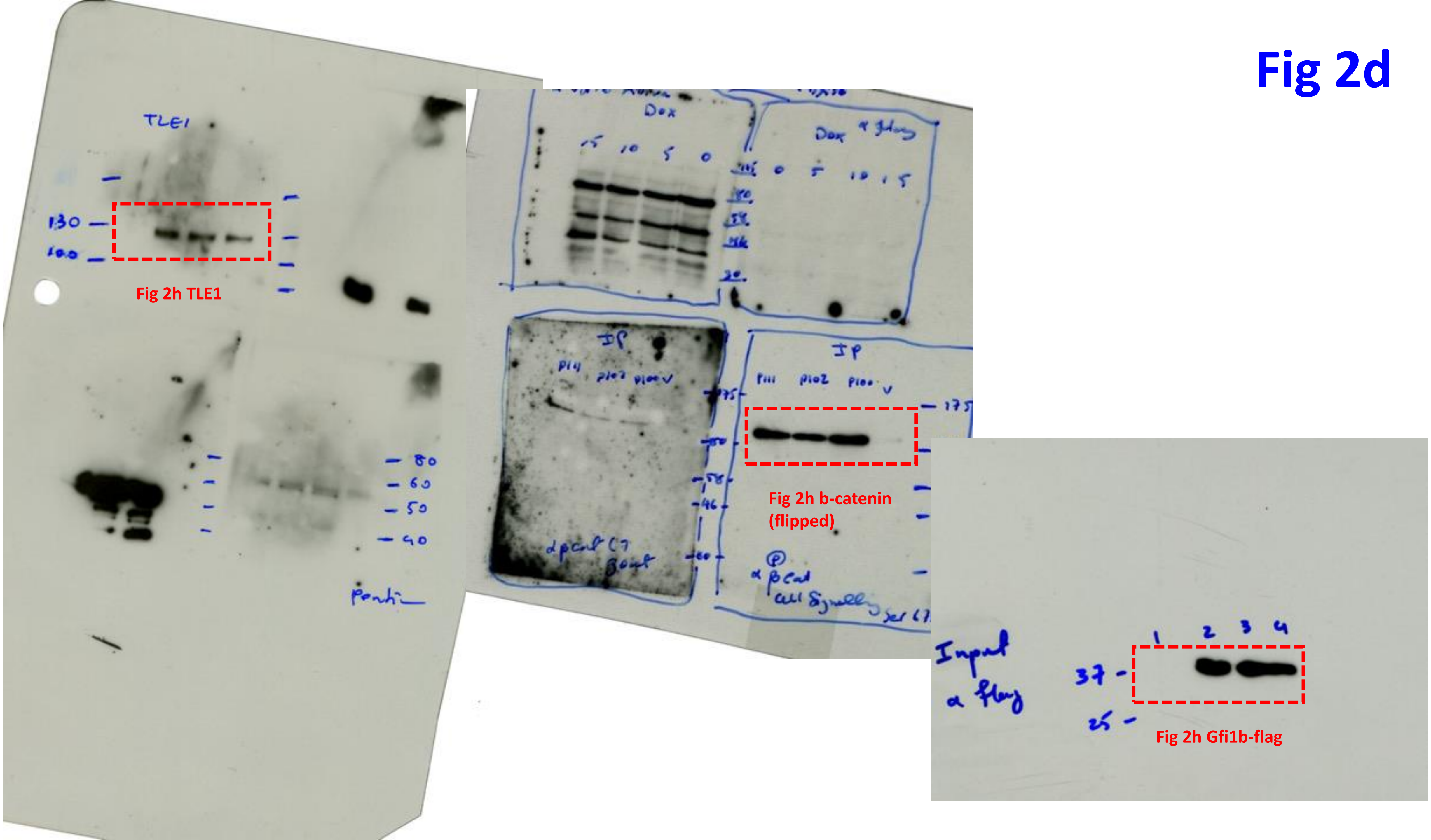
TLE1
130
100
Fig 2h TLE1
80
60
50
40
Dox
15 10 5 0
IP
P111 P102 P100 V
175
Fig 2h b-catenin
(flipped)
Input
α flag
1 2 3 4
37
25
Fig 2h Gfi1b-flag

Fig 2e

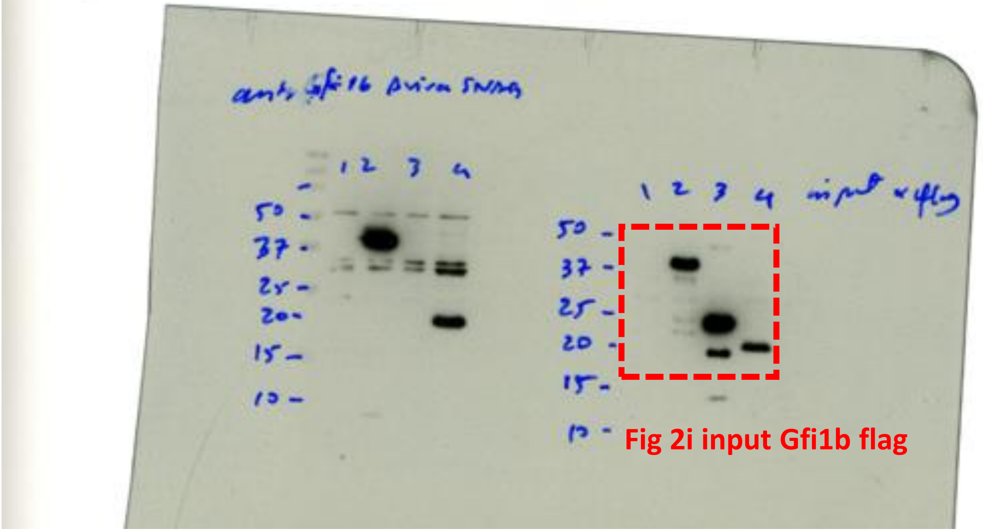
Fig 2i input Gfi1b flag

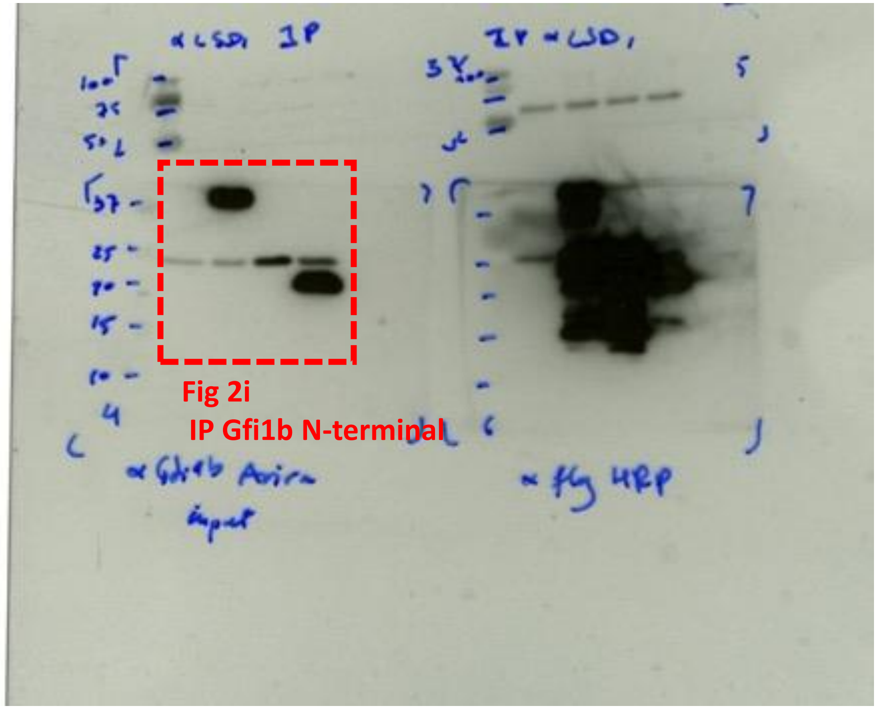
Fig 2i
IP Gfi1b N-terminal

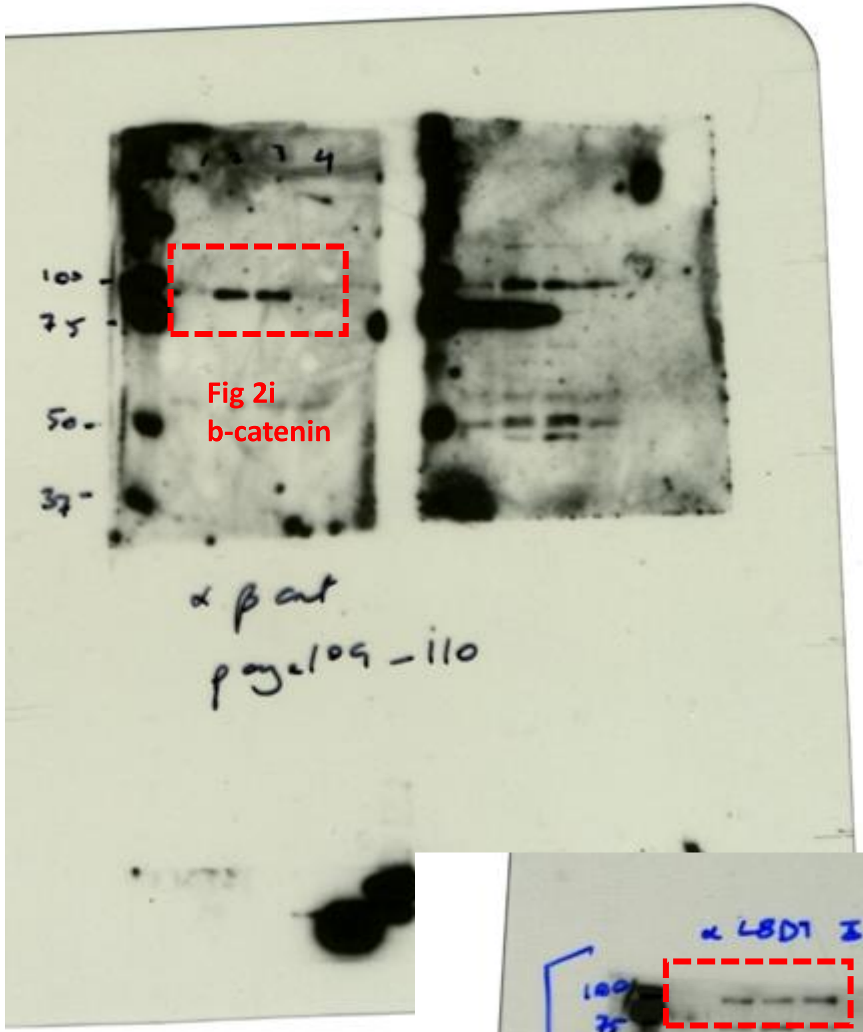
Fig 2i
b-catenin

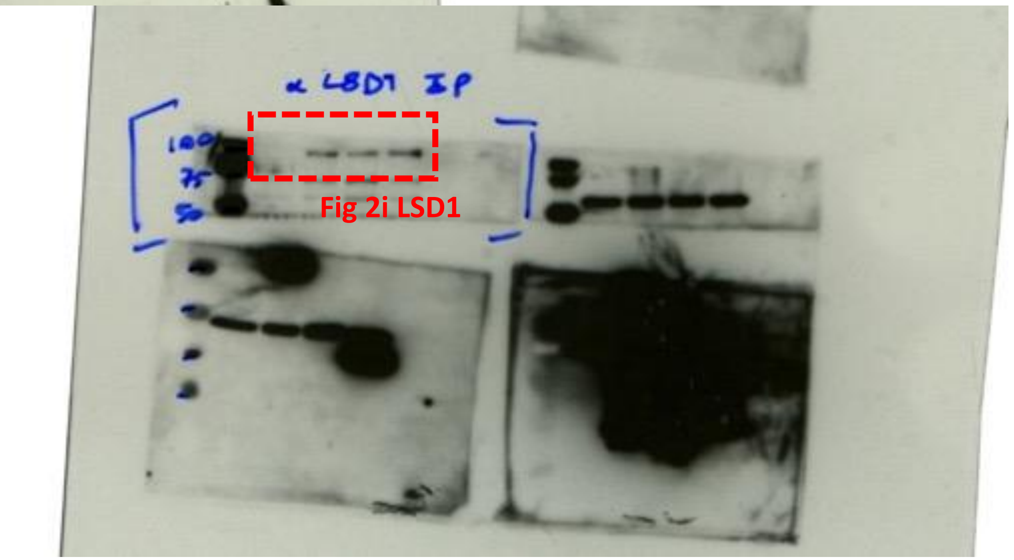
Fig 2i LSD1

Fig 2f

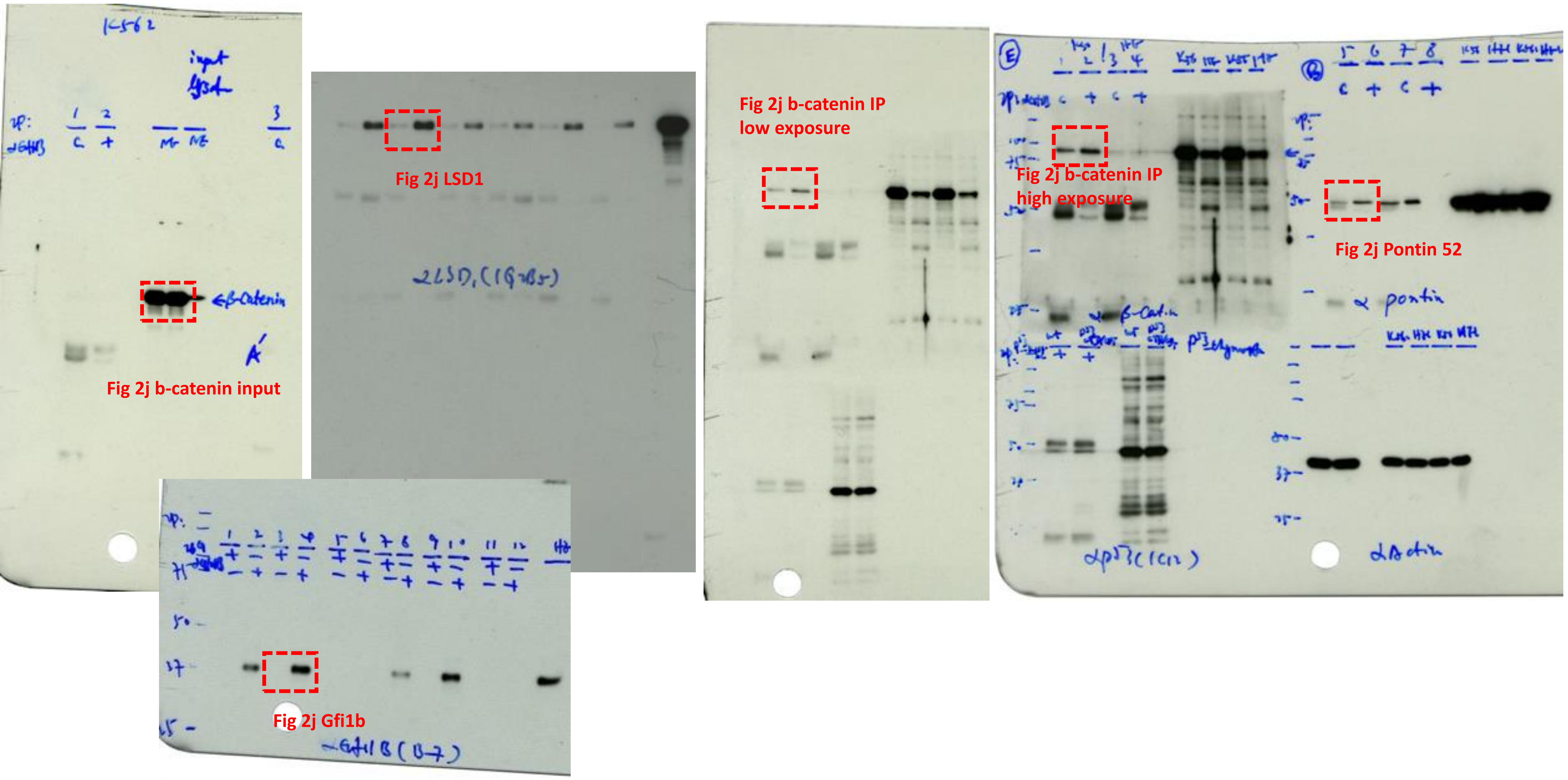
Fig 2j LSD1
Fig 2j b-catenin IP low exposure
Fig 2j b-catenin IP high exposure
Fig 2j Pontin 52
Fig 2j b-catenin input
Fig 2j Gfi1b

Fig 2g

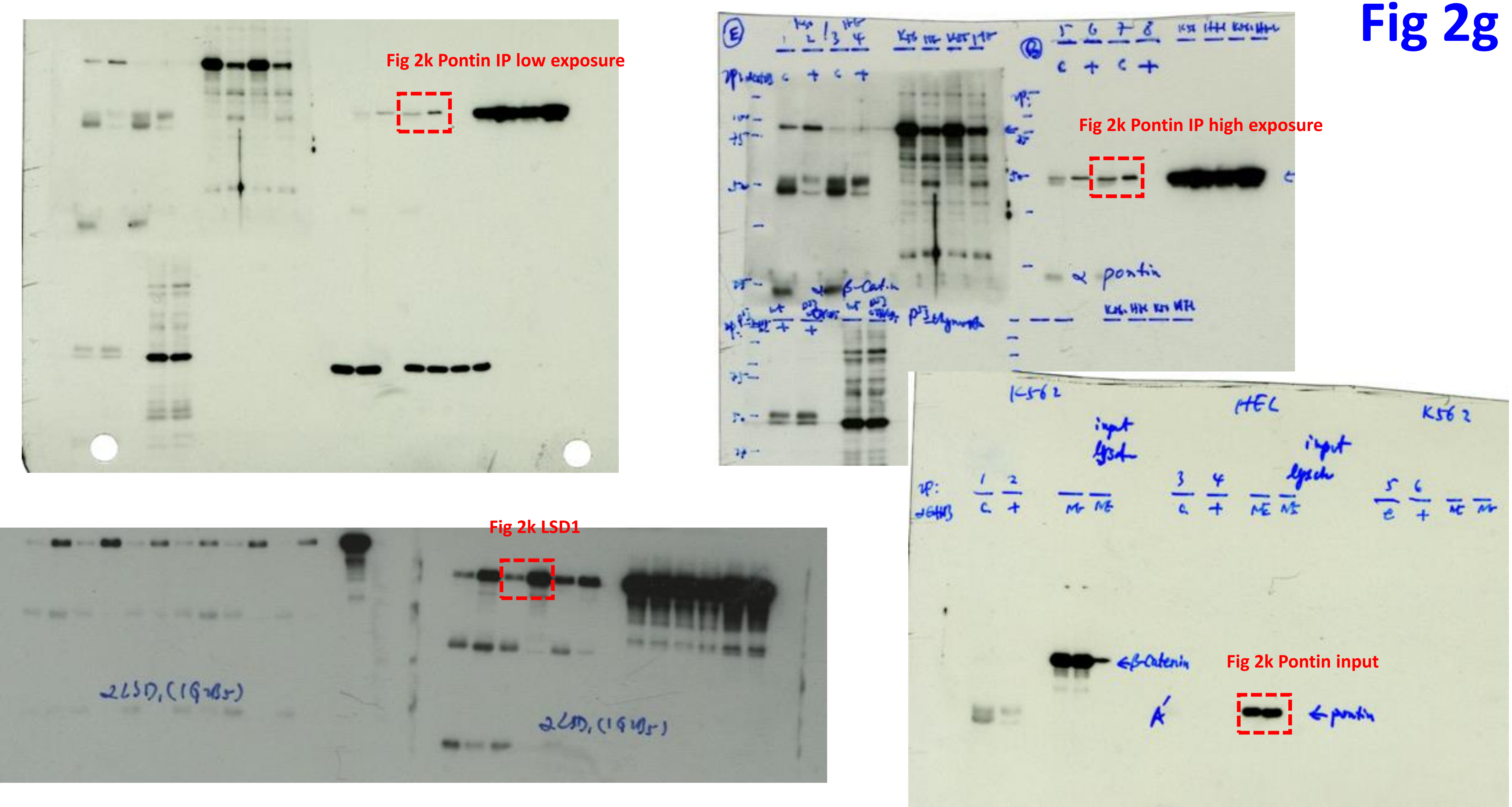
Fig 2k Pontin IP low exposure
Fig 2k Pontin IP high exposure
Fig 2k LSD1
Fig 2k Pontin input
K562
HEL
K562
←β-Catenin
←pontin
α pontin

Fig 2i

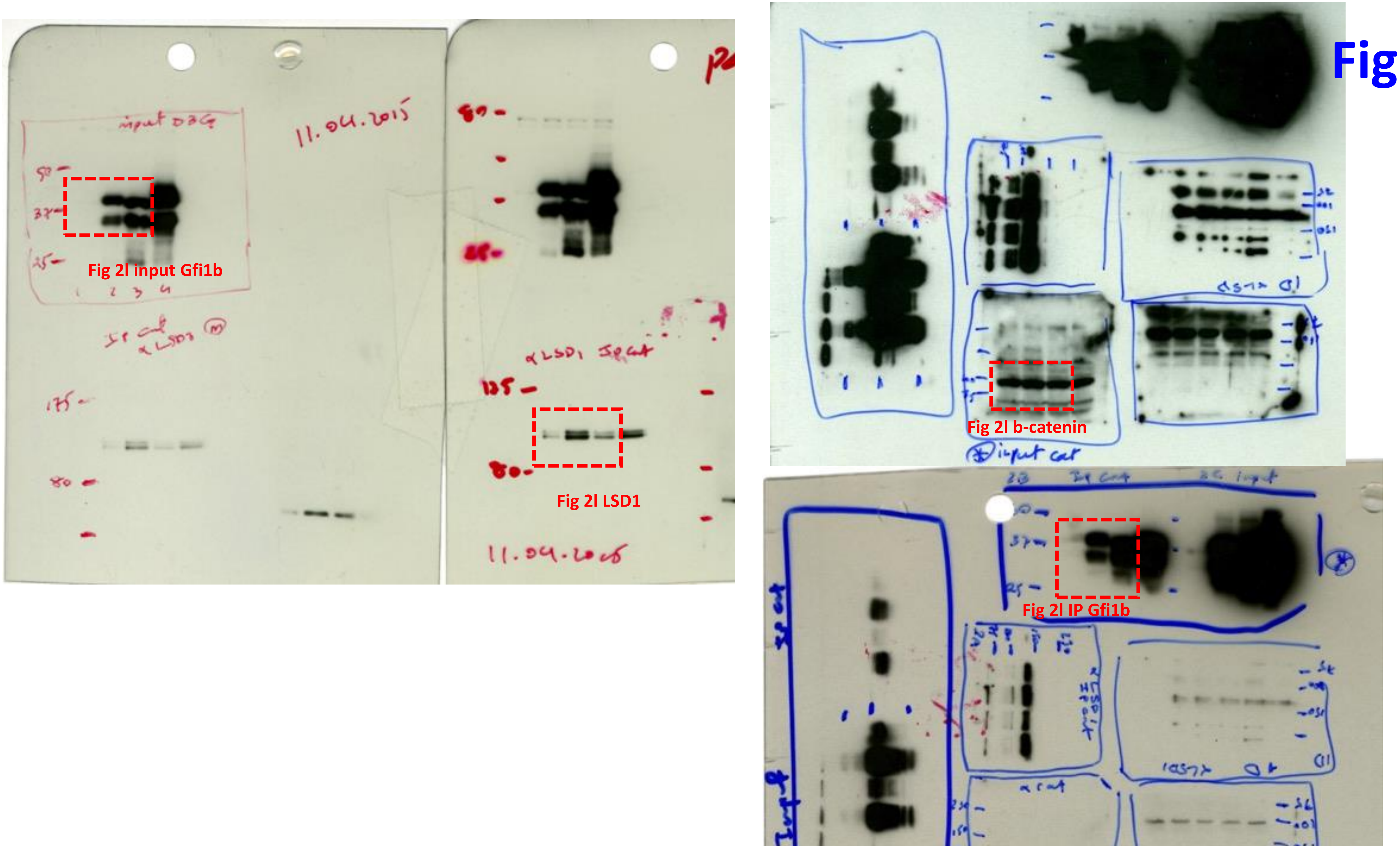
Fig 2l input Gfi1b
Fig 2l LSD1
Fig 2l b-catenin
Fig 2l IP Gfi1b
11.04.2015

Fig 2j

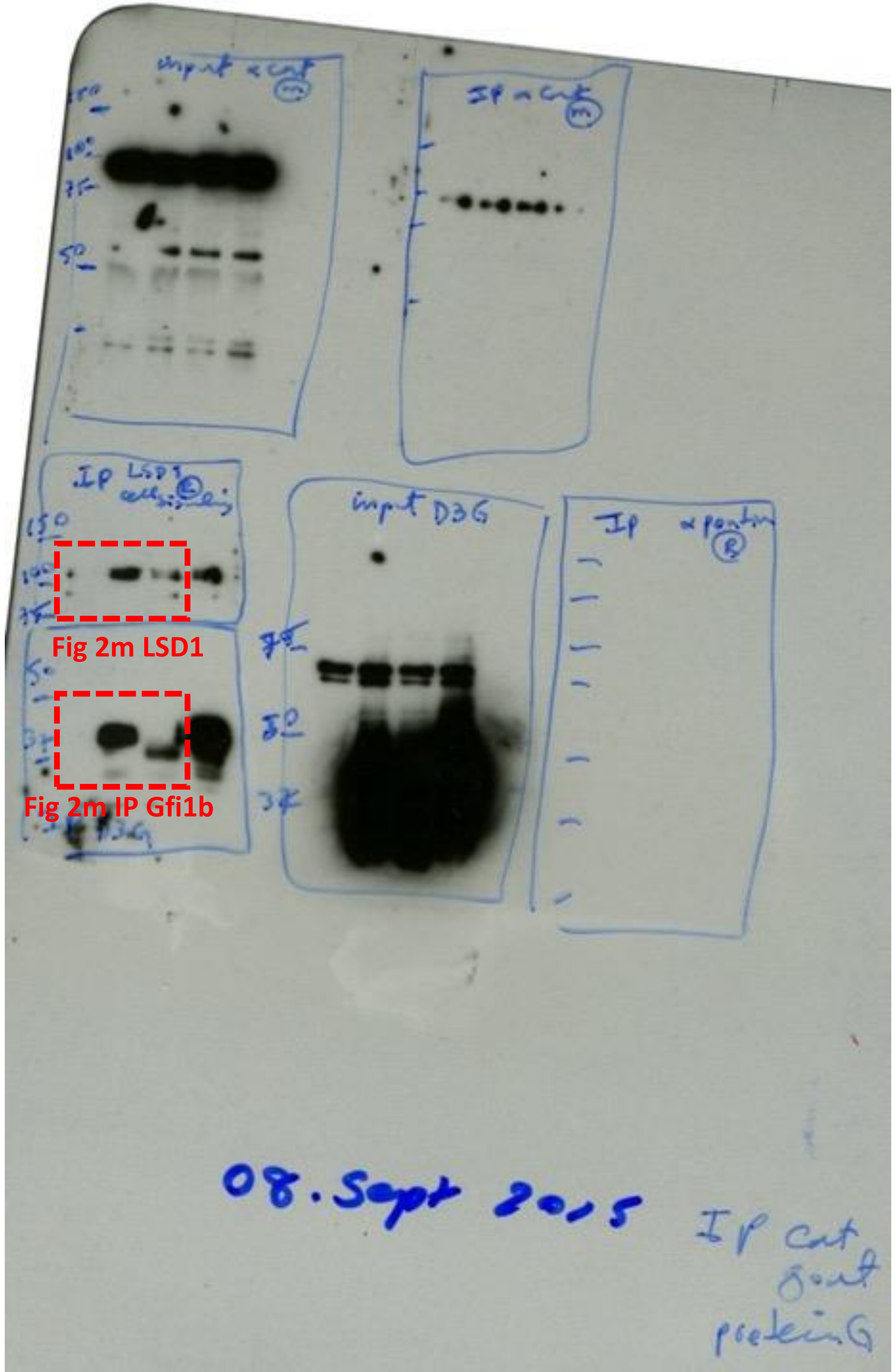
Fig 2m LSD1
Fig 2m IP Gfi1b
input D3G
08. Sept 2015
IP cat
Goat
proteinG

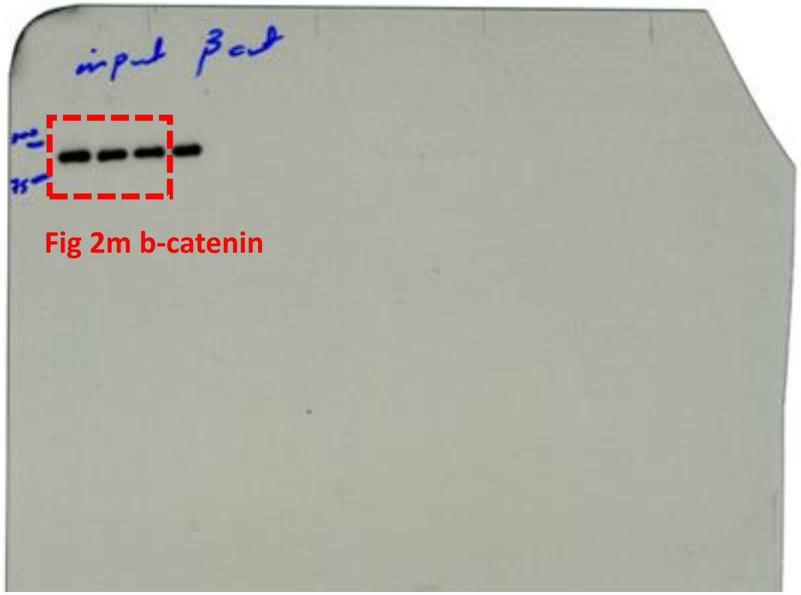
input βcat
Fig 2m b-catenin

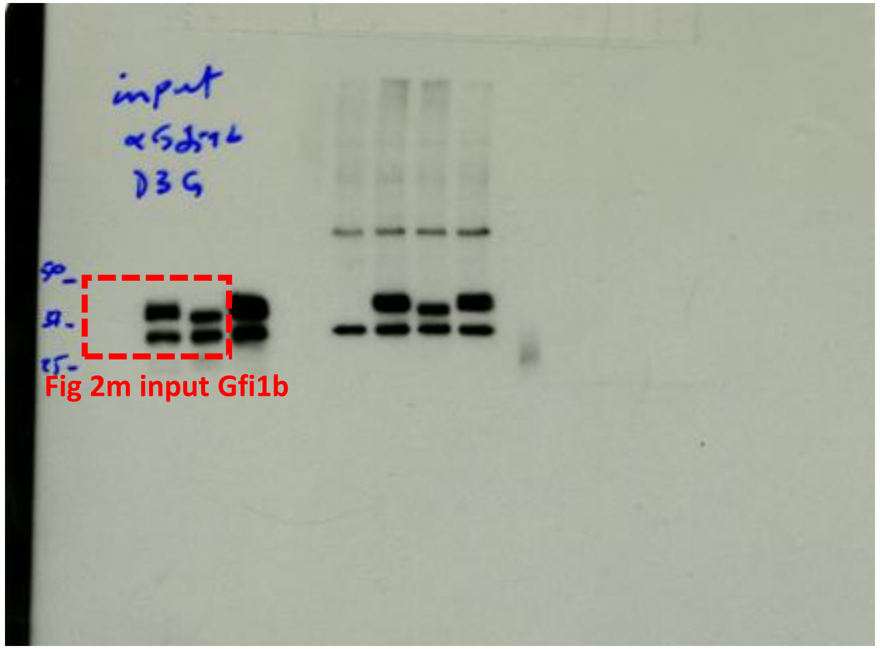
input
D3G
Fig 2m input Gfi1b

Fig 2k

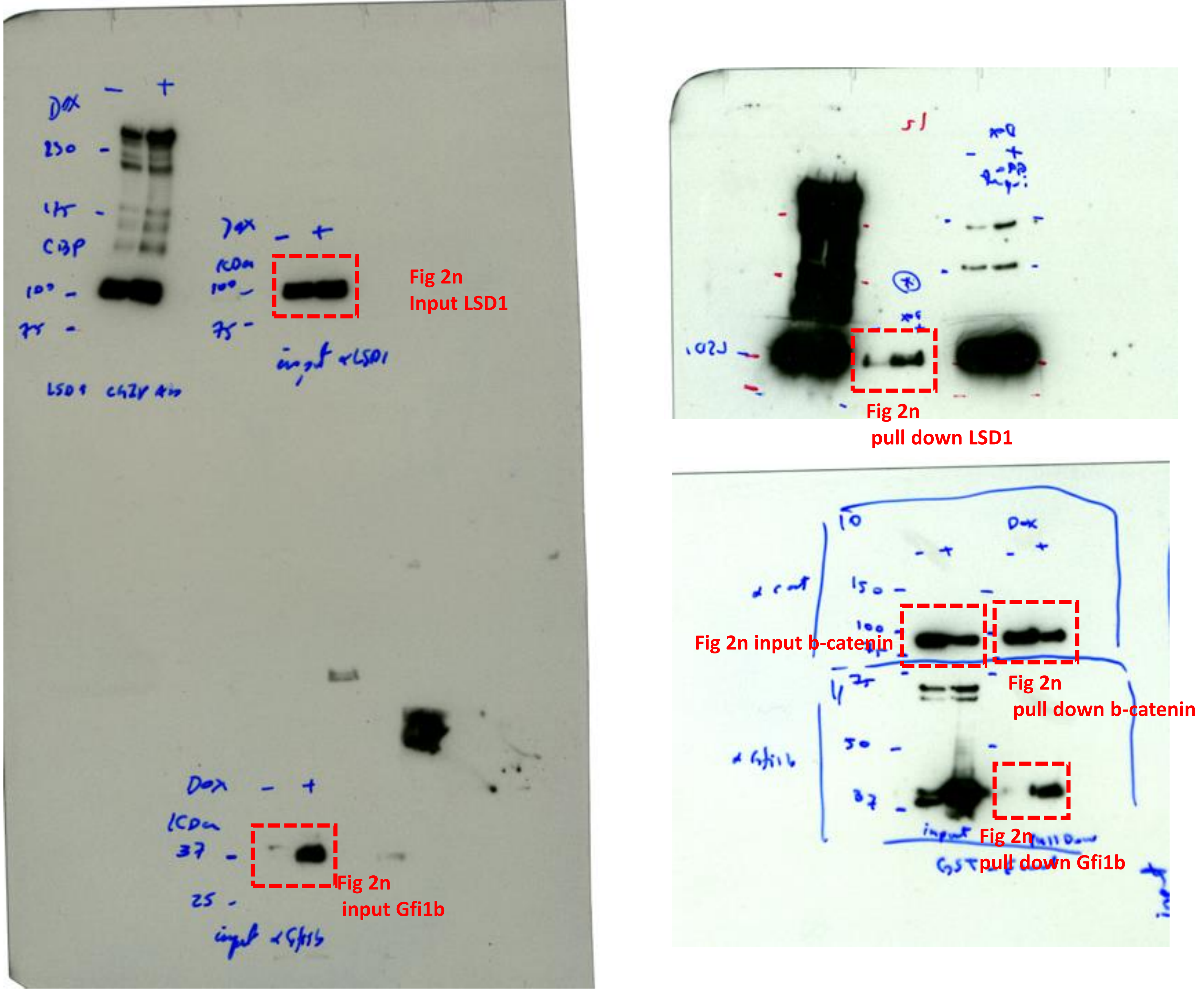
Fig 2n
Input LSD1
Fig 2n
input Gfi1b
Fig 2n
pull down LSD1
Fig 2n input b-catenin
Fig 2n
pull down b-catenin
Fig 2n
pull down Gfi1b

Fig 2h

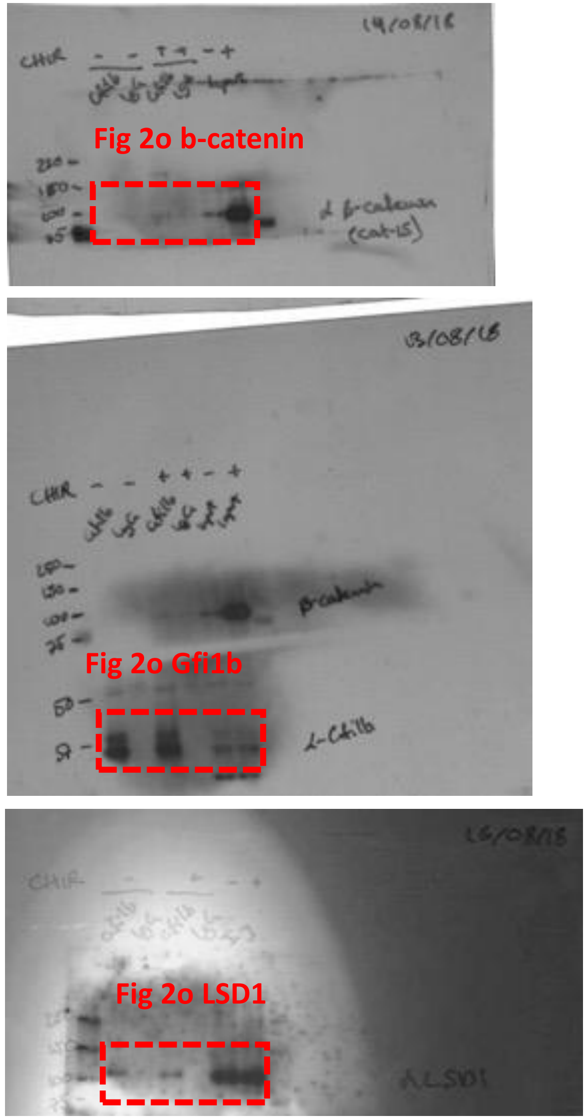

Fig 3l

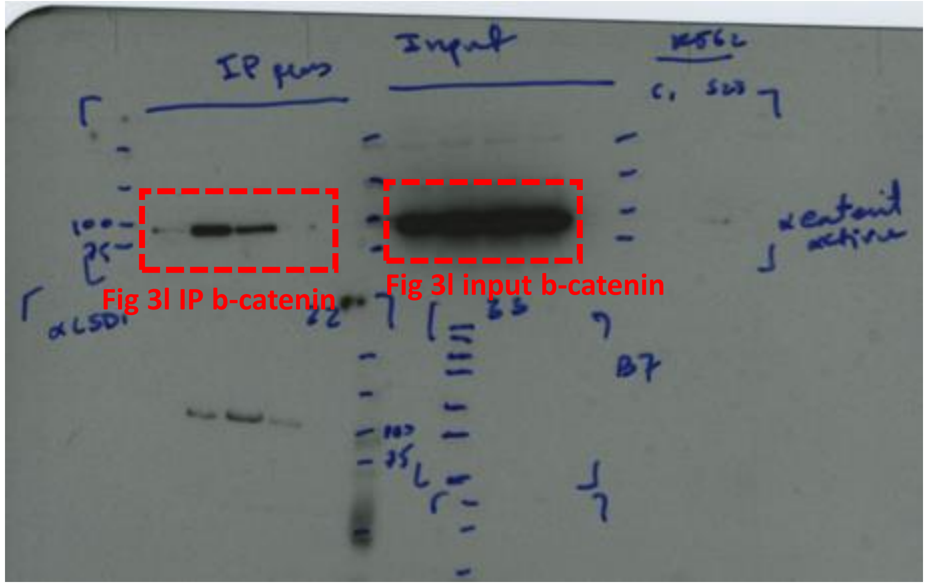
Fig 3l IP b-catenin
Fig 3l input b-catenin

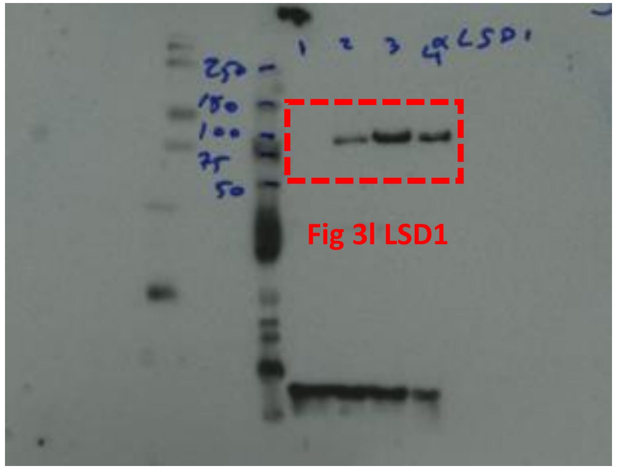
Fig 3l LSD1

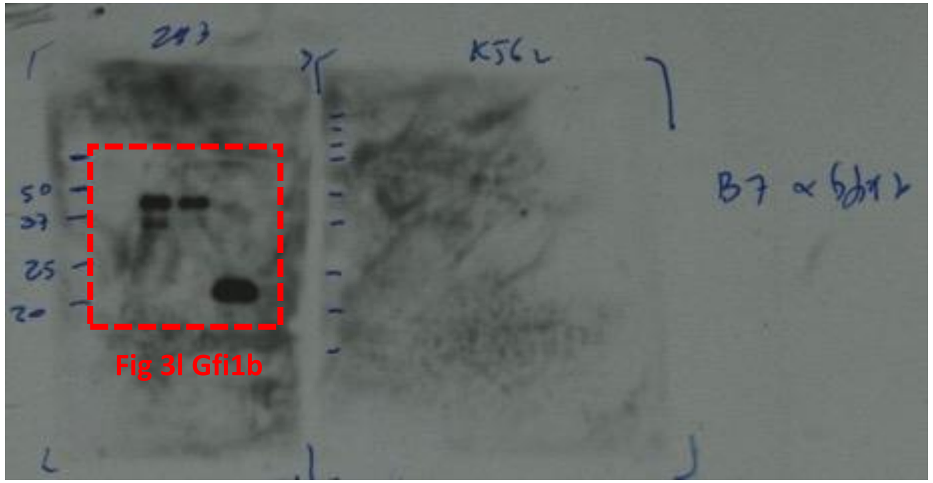
Fig 3l Gfi1b

Fig 5e

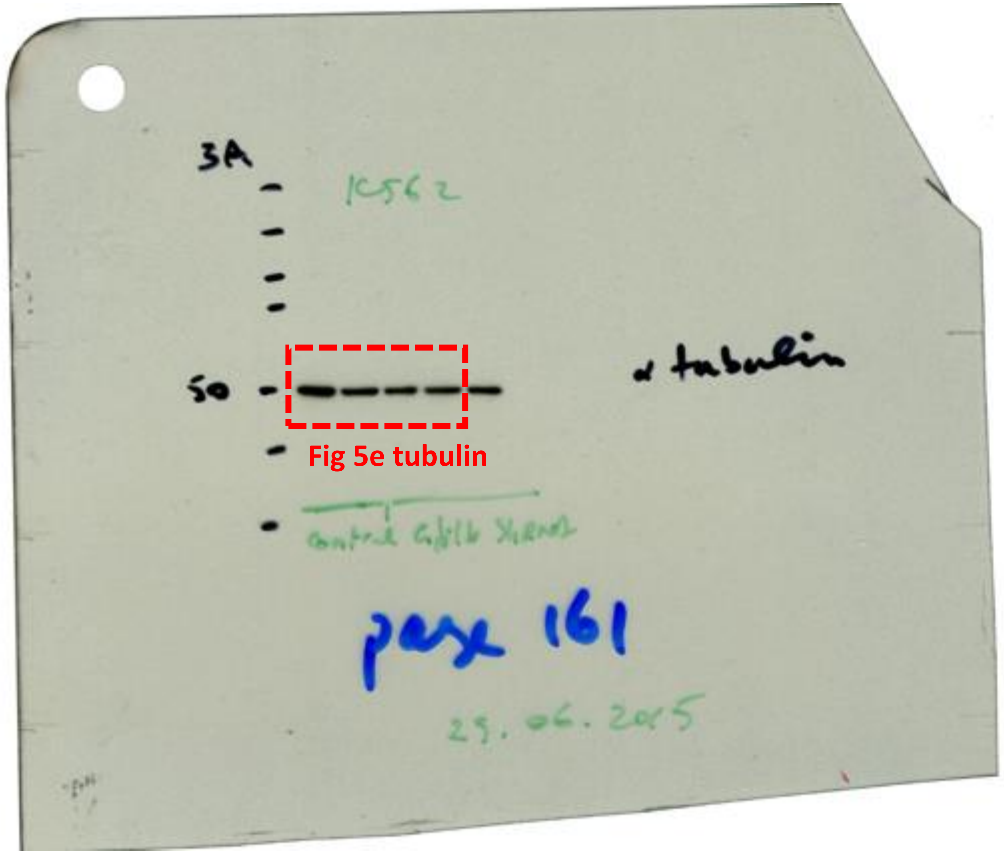
3A
K562
50
α tubulin
Fig 5e tubulin
page 161
29.06.2015

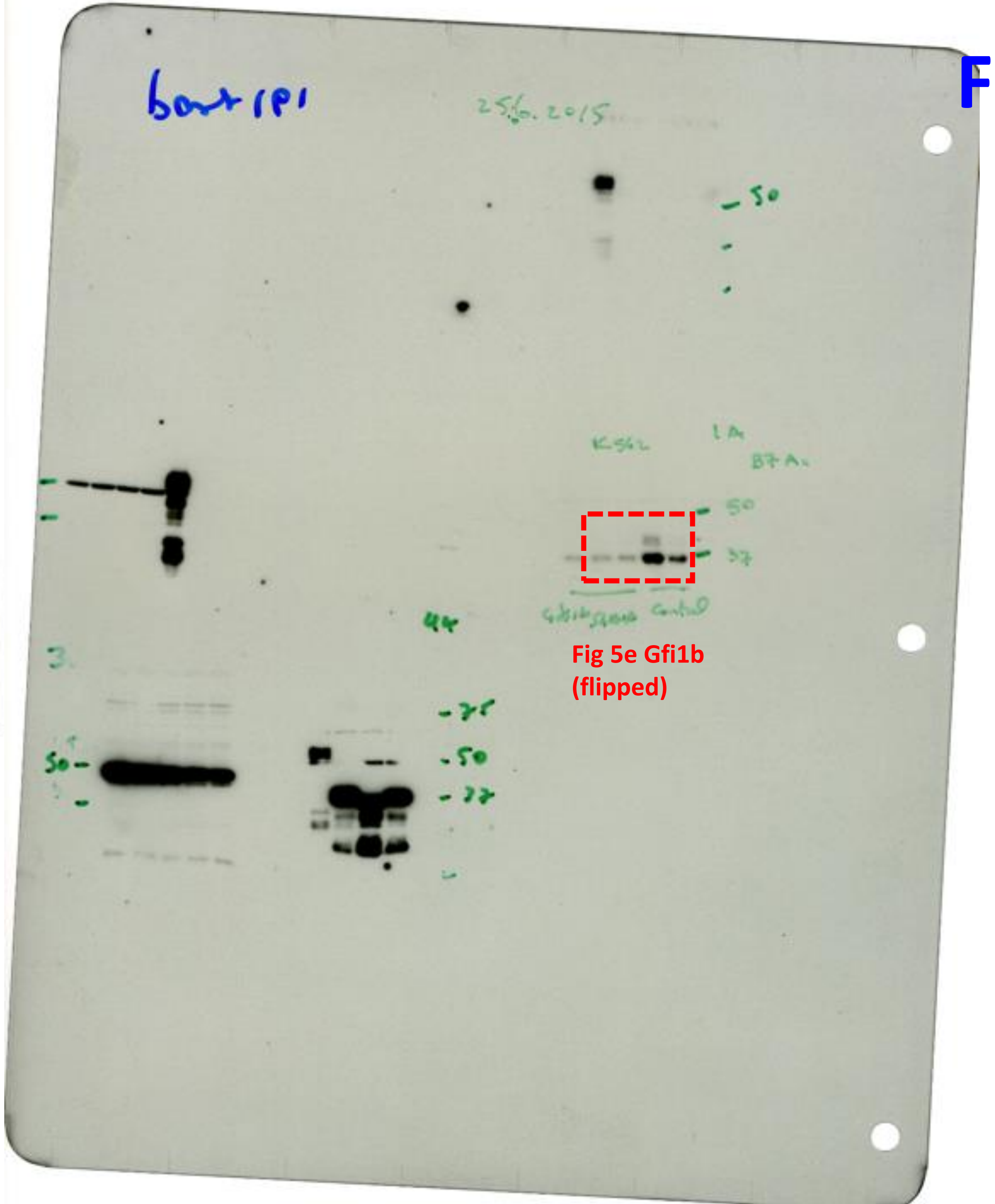
25.6.2015
50
K562
50
37
Fig 5e Gfi1b
(flipped)
75
50
37

Suppl Fig 2a

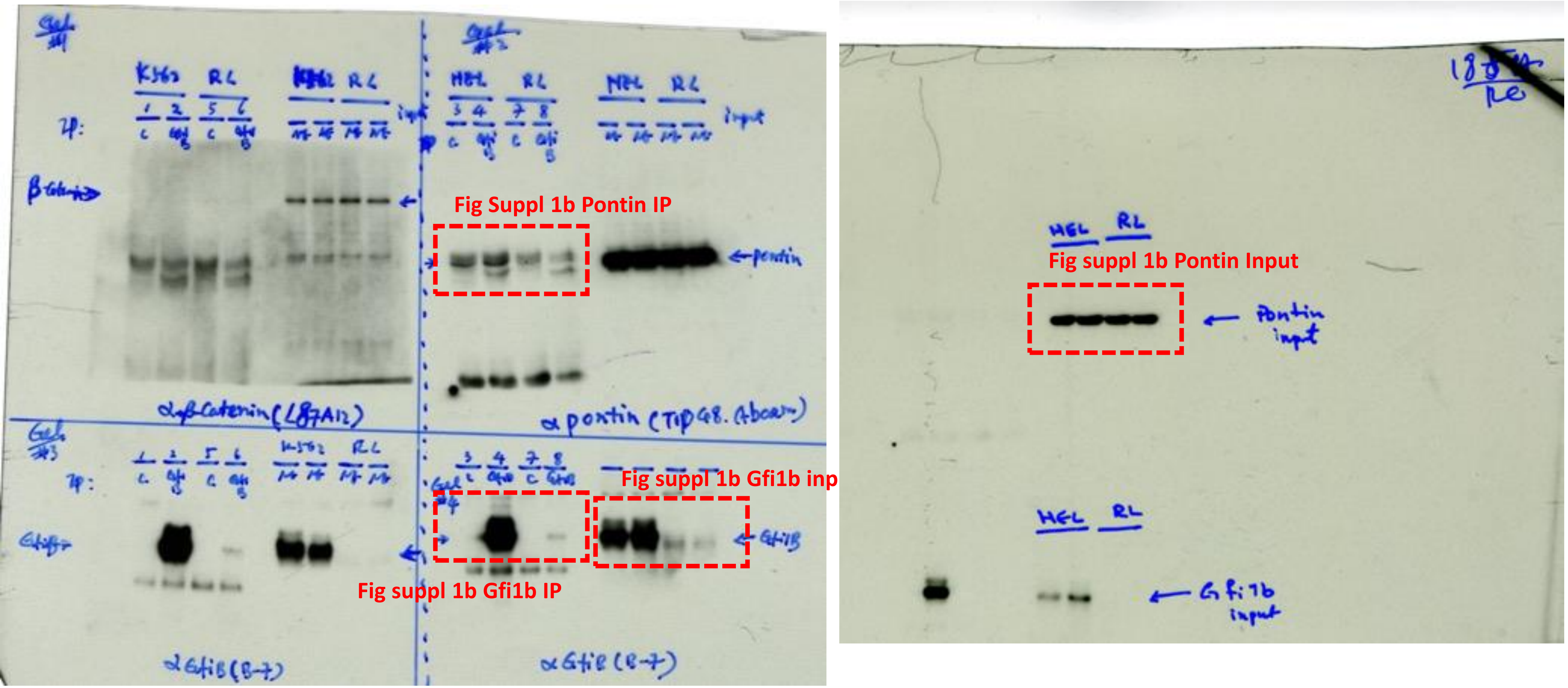
Fig Suppl 1b Pontin IP
Fig suppl 1b Gfi1b inp
Fig suppl 1b Gfi1b IP
Fig suppl 1b Pontin Input
HEL
RL
Pontin input
Gfi1b input

Suppl Fig 2c

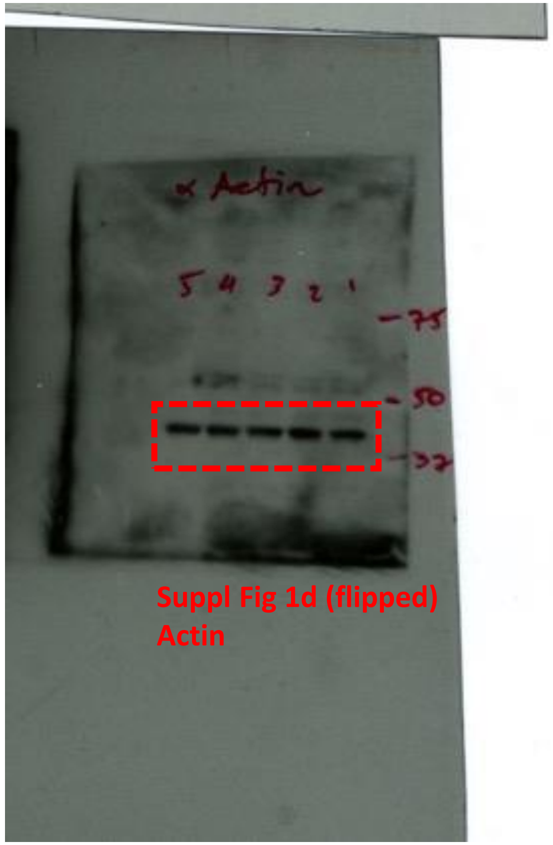

Suppl Fig 1d (flipped)
Actin

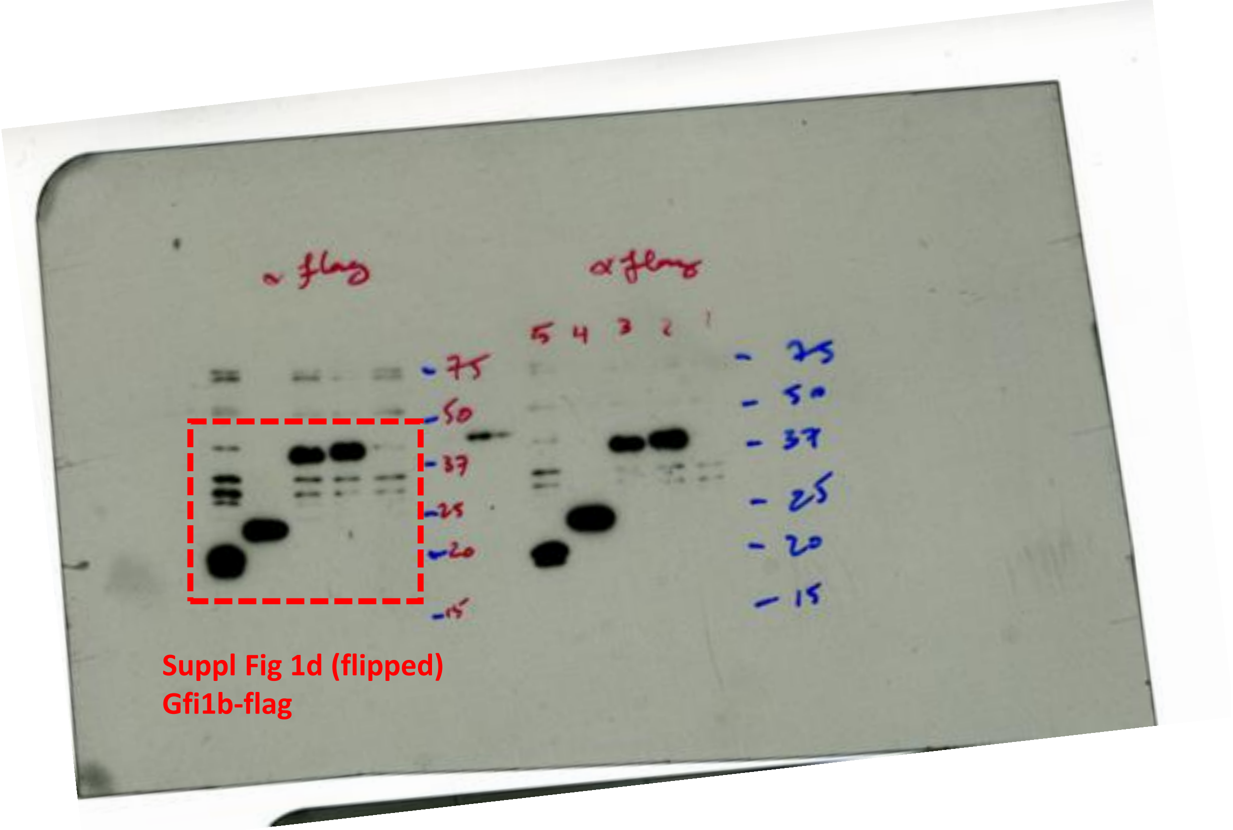

Suppl Fig 1d (flipped)
Gfi1b-flag

Suppl Fig 6

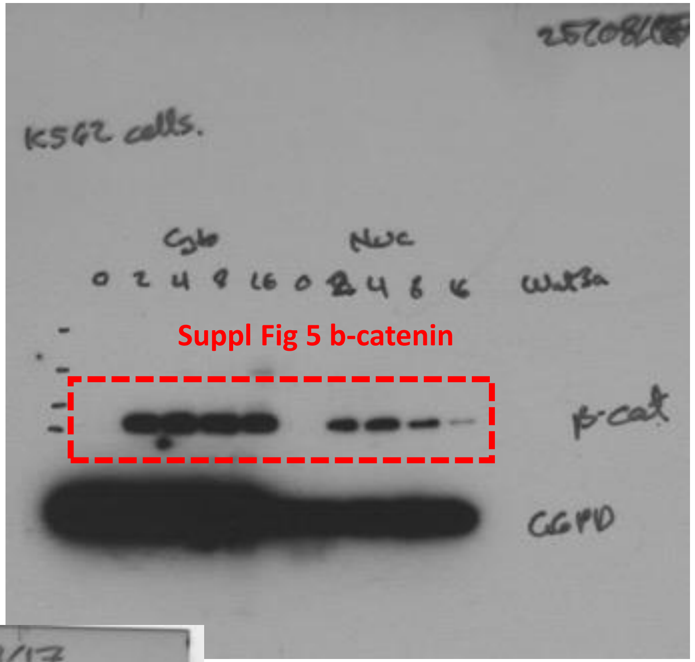
K562 cells.
Suppl Fig 5 b-catenin
β-cat
G6PD

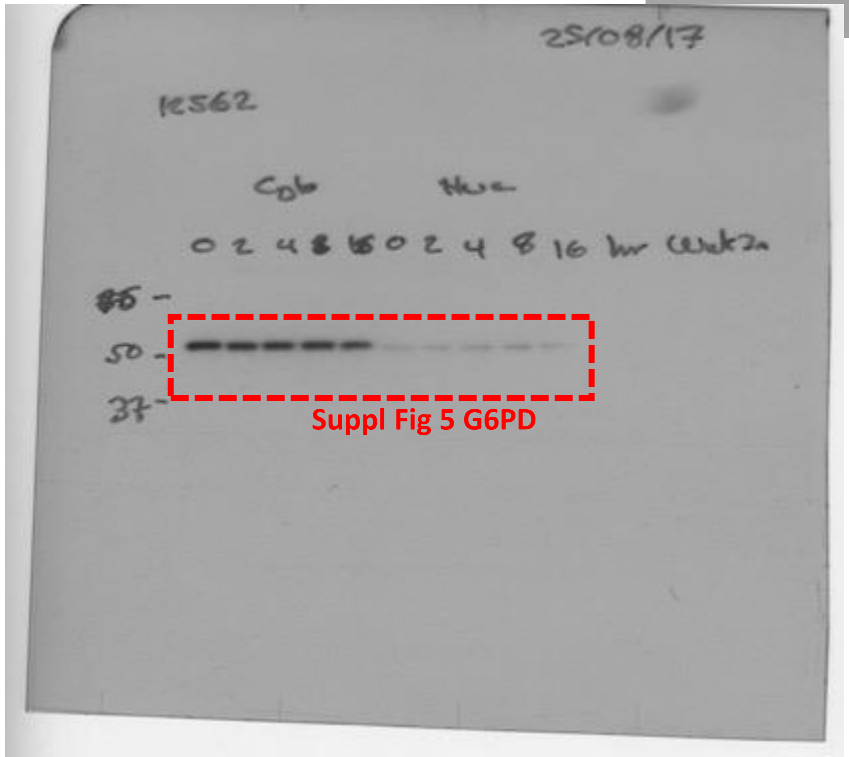
25/08/17
K562
Suppl Fig 5 G6PD

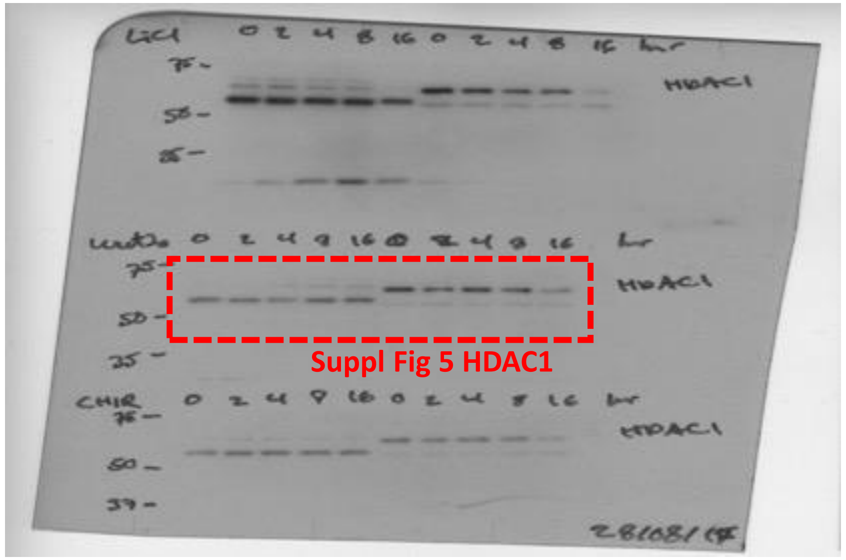
HDAC1
Suppl Fig 5 HDAC1
HDAC1
HDAC1

Supplementary Figure 9: Uncropped and unprocessed scans of western blots.

Supplementary Table 1		
Sample	total number of reads	uniquely mapped
k562_h3k4me1_nownt1	25022437	20144080
k562_h3k4me1_nownt2	26369037	21208732
k562_h3k4me1_nownt3	26359923	21186662
k562_h3k4me1_wnt1	27924278	23644354
k562_h3k4me1_wnt2	26200328	22190501
k562_h3k4me1_wnt3	27700026	23440462
k562_h3k9me2_nownt1	27803372	23490680
k562_h3k9me2_nownt2	26041154	22004891
k562_h3k9me2_nownt3	27731173	23415096
k562_h3k9me2_wnt1	30000720	23990723
k562_h3k9me2_wnt2	29995486	24006466
k562_h3k9me2_wnt3	28381687	22726595
k562_h3_nownt1	30635981	24722006
k562_h3_nownt2	30698192	24790726
k562_h3_nownt3	29141934	23548877
k562_h3_wnt1	28719475	23228954
k562_h3_wnt2	26989688	21839831
k562_h3_wnt3	28696587	23192652
bcat_negwnt3a_k652_1	30278357	23856365
bcat_negwnt3a_k652_2	30252789	23827307
bcat_negwnt3a_k652_3	30557638	24057147
bcat_poswnt3a_k652_1	33113078	26153388
bcat_poswnt3a_k652_2	33070093	26110840
bcat_poswnt3a_k652_3	33395664	26357755
gfi1b_negwnt3a_k652_1	33439181	26360403
gfi1b_negwnt3a_k652_2	33542289	26446165
gfi1b_negwnt3a_k652_3	33887317	26709938
gfi1b_poswnt3a_k652_1	35656335	28023486
gfi1b_poswnt3a_k652_2	35607881	27976817
gfi1b_poswnt3a_k652_3	36009188	28288322
input_negwnt3a_1	34147910	28013067
input_negwnt3a_2	34463978	28264241
input_negwnt3a_3	34072210	27944362
input_poswnt3a_1	35490751	29162353
input_poswnt3a_2	35830439	29436526
input_poswnt3a_3	35452587	29125706
lsd1_negwnt3a_k652_1	35441162	28091909
lsd1_negwnt3a_k652_2	35155023	27879997

lsd1_negwnt3a_k652_3	35094745	27828170
lsd1_poswnt3a_k652_1	37932502	30216123
lsd1_poswnt3a_k652_2	38253033	30459060
lsd1_poswnt3a_k652_3	37880962	30171928

Supplementary Table 1 : RNA-seq total read numbers and aligned read numbers

Supplementary Table 2
Nusse targets up: adapted from Roel Nusse's Wnt targets website (http://web.stanford.edu/group/nusselab/cgi-bin/wnt/target_genes)
Labbe wnt3a up: PMID: 17210685
Labbe Wnt3a down: PMID: 17210685
Willert_wnt_signaling: PMID: 12095419
Kolligs targets: PMID: 24467841
GO canonical wnt: adapted from GO ontology, Gene Ontology Consortium (http://www.geneontology.org)
GO wnt: adapted from GO ontology, Gene Ontology Consortium (http://www.geneontology.org)
KEGG wnt: KEGG Pathway database (https://www.genome.jp/kegg/pathway.html)
Canonical Wnt targets: adapted from PMID: 17210685 , PMID: 24889652 and Roel Nusse's Wnt targets website (http://web.stanford.edu/group/nusselab/cgi-bin/wnt/target_genes)
Non canonical Wnt targets: adapted from PMID: 29453334

Supplementary Table 2 : References of Gene sets used in GSEA

Supplementary Table 3		
**Figure 1g**	Gfi1b wt/flox	n = 70
	Gfi1b flox/flox	n = 220
**Figure 3a**	n =3 for each data point	
**Figure 3b**	n =3 for each data point	
**Figure 3c**	n =3 for each data point	
**Figure 3d**	n =3 for each data point	
**Figure 3e**	n =3 for each data point	
**Figure 3f**	n =3 for each data point	
**Figure 3g**	n =3 for each data point	
**Figure 3h**	n =3 for each data point	
**Figure 3i**	n =3 for each data point	
**Figure 3j**	n =3 for each data point	
**Figure 3k**	n =3 for each data point	
**Figure 4e**	Gfi1b WT	n = 5
	GFI1b KO	n = 5
**Figure 5f**	n =3 for each data point	
**Figure 5g**	n =3 for each data point	
**Figure 7d**	n =3 for each data point	
**Figure 8d**	Gfi1b wt/flox wnt3a = 0	n = 39
	Gfi1b flox/flox wnt3a = 0	n = 46
	Gfi1b flox/flox wnt3a = 0.9	n = 95
	Gfi1b flox/flox wnt3a = 1.3	n = 83
	Gfi1b flox/flox wnt3a = 1.9	n = 91
	Gfi1b flox/flox wnt3a = 2.5	n = 111
	Gfi1b flox/flox wnt3a = 3.1	n = 87
	Gfi1b wt/flox wnt3a = 0.9	n = 120
	Gfi1b wt/flox wnt3a = 1.3	n = 89
	Gfi1b wt/flox wnt3a = 1.9	n = 108
	Gfi1b wt/flox wnt3a = 2.5	n = 86
	Gfi1b wt/flox wnt3a = 3.1	n = 81
**Supplementary 2b**	n =3 for each data point	
**Supplementary 8b**	b-catenin	n = 3

	GFP	n = 2
**Supplementary 8c**	Gfi1b wt/flox wnt3a = 0	n = 39
**(Same as figure 8d)**	Gfi1b wt/flox wnt3a = 0.9	n = 120
	Gfi1b wt/flox wnt3a = 1.3	n = 89
	Gfi1b wt/flox wnt3a = 1.9	n = 108
	Gfi1b wt/flox wnt3a = 2.5	n = 86
	Gfi1b wt/flox wnt3a = 3.1	n = 81
	Gfi1b flox/flox wnt3a = 0	n = 46
	Gfi1b flox/flox wnt3a = 0.9	n = 95
	Gfi1b flox/flox wnt3a = 1.3	n = 83
	Gfi1b flox/flox wnt3a = 1.9	n = 91
	Gfi1b flox/flox wnt3a = 2.5	n = 111
	Gfi1b flox/flox wnt3a = 3.1	n = 87

Supplementary Table 3 : The sample size of data points for each assay

Supplementary Table 4

Primers used for ChIP-PCR

Gene	Forward Primer (5'-3')	Reverse Primer (5'-3')
Axin2	GGCTGCGCTTTGATAAGGTC	CCCGAAATCCATCGCTCTGA
Bambi	GATCGCCACTCCAGCTACATC	CTTTGGTGAGCAGCACGG
Birc5	CTCCCTGCTTTGTCCCCATC	CATCTGCAAGGGACAGCACA
Ccnd1	TCTGCCGGGCTTTGATCTTT	GGCTCCAGGACTTTGCAACT
CCRL2	CAGGGAAATCAAAGGCGGGG	TCCAGAGGATCTCTGAAGCG
Cdh1	CTCCCACCCCAATCTGAACC	CCTTCAGGCAGTCTTGTCCC
Gapdh	CCACATCGCTCAGACACCAT	CCCGCAAGGCTCGTAGAC
Intergenic control	CCTGGCCTCTCACACTCA	AGAACCCTTGCTCTCCAC
Nfat5	TGCCCTCGGACTTCATCTCAT	CAGATTCTCGCGAGTAGAGGG
Ptk7	CTCCTTTTCCTGAGCCCGC	CAACTCTCGGGTACTCACCG
Rock2	GTTAGTGTCTAAGCCGGGCA	ATTCCGAATTAGCGGAGGCG
SLC38A8	GTCGGGGGTTGAGACCATTAG	GTCCTTATCTGTCCGACACCA
Sp5	TTGATGATTGGGTAGCGGCA	TAAGGACTTTGCTGGGCCG
Sp8	GTGGAATGGGCGGAACTGA	ACACAAAAGTGCCCTCCTCC
Yaf2	GCGTTTGAAGTCTCCGGC	AGCTGTCGGGGAAACTCCT
Zfp467	CTCTCCTCCCCGCGAAGTT	GCTCACAATGGCGCTCG

Primers used for q-PCR

Gene	Forward Primer (5'-3')	Reverse Primer (5'-3')
CCND1	ATGCCAACCTCCTCAACGAC	TCCTCCTCGCACTTCTGTTC
NFAT5	GACCCTGACAACTATTCAAACC	TGCTGTAAAGTCTGTGCTTG
BIRC5	ACGCCTGTAATACCAGCAC	GCTCTTTCTCTGTCCAGTTTC
PTK7	AGCGTGGAGGTGTATGATG	AAGTGTAGTTGCCAGCGTC
ROCK2	ACTTGGGAGAAATGGGGTGG	AGGTAAATCCGATGAAAGGC

Supplementary Table 4 : Primers' list
