# Supplementary material for: Gfi1b regulates the level of Wnt/β-catenin signaling in hematopoietic stem cells and megakaryocytes
Source: Nat Commun. 2019 Mar 20;10:1270. doi: 10.1038/s41467-019-09273-z (PMC6426870; doi:10.1038/s41467-019-09273-z)
Supplement: Supplementary file 3 — Reporting Summary [file 41467_2019_9273_MOESM3_ESM.pdf]

nature research

Corresponding author(s): *Double-blind peer review submissions: write DBPR and your manuscript number here instead of author names.*

Reporting Summary

Nature Research wishes to improve the reproducibility of the work that we publish. This form provides structure for consistency and transparency in reporting. For further information on Nature Research policies, see Authors & Referees and the Editorial Policy Checklist.

Statistical parameters

When statistical analyses are reported, confirm that the following items are present in the relevant location (e.g. figure legend, table legend, main text, or Methods section).

n/a	Confirmed	
☐	☒	The exact sample size (*n*) for each experimental group/condition, given as a discrete number and unit of measurement
☐	☒	An indication of whether measurements were taken from distinct samples or whether the same sample was measured repeatedly
☐	☒	The statistical test(s) used AND whether they are one- or two-sided<br>*Only common tests should be described solely by name; describe more complex techniques in the Methods section.*
☒	☐	A description of all covariates tested
☒	☐	A description of any assumptions or corrections, such as tests of normality and adjustment for multiple comparisons
☒	☐	A full description of the statistics including central tendency (e.g. means) or other basic estimates (e.g. regression coefficient) AND variation (e.g. standard deviation) or associated estimates of uncertainty (e.g. confidence intervals)
☒	☐	For null hypothesis testing, the test statistic (e.g. *F*, *t*, *r*) with confidence intervals, effect sizes, degrees of freedom and *P* value noted<br>*Give P values as exact values whenever suitable.*
☒	☐	For Bayesian analysis, information on the choice of priors and Markov chain Monte Carlo settings
☒	☐	For hierarchical and complex designs, identification of the appropriate level for tests and full reporting of outcomes
☒	☐	Estimates of effect sizes (e.g. Cohen's *d*, Pearson's *r*), indicating how they were calculated
☐	☒	Clearly defined error bars<br>*State explicitly what error bars represent (e.g. SD, SE, CI)*

Our web collection on statistics for biologists may be useful.

Software and code

Policy information about availability of computer code

Data collection	Not applicable
Data analysis	Scaffold (ver4.8.2) was used to analyse mass spectrometry data. bStatistical analysis was done with Graph-Pad Prism software (GraphPad software, La Jolla, CA, USA).

For manuscripts utilizing custom algorithms or software that are central to the research but not yet described in published literature, software must be made available to editors/reviewers upon request. We strongly encourage code deposition in a community repository (e.g. GitHub). See the Nature Research guidelines for submitting code & software for further information.

Data

Policy information about availability of data

All manuscripts must include a data availability statement. This statement should provide the following information, where applicable:

- Accession codes, unique identifiers, or web links for publicly available datasets
- A list of figures that have associated raw data
- A description of any restrictions on data availability

The raw proteomics data, which are presented in Figure 2a-b and 2l, will be uploaded to the PRIDE archive (https://www.ebi.ac.uk/pride/archive/) and be available under the following

accession number: [MassIVE ID is MSV000083125].
The raw ChIP-seq and RNA-seq data, which is presented in Figures 5-7 and Supplementary figures 3 and 7, have been uploaded to the GEO Datasets repository (https://www.ncbi.nlm.nih.gov/gds) and is available under the following accession numbers: GSE71310, GSE85737 and GSE117944.
Previously published ChIP-seq data, which is presented in Supplementary figures 4 and 5, is available under the following accession numbers: GSE43597, GSE22557.

Field-specific reporting

Please select the best fit for your research. If you are not sure, read the appropriate sections before making your selection.

- [x] Life sciences
- [ ] Behavioural & social sciences
- [ ] Ecological, evolutionary & environmental sciences

For a reference copy of the document with all sections, see nature.com/authors/policies/ReportingSummary-flat.pdf

Life sciences study design

All studies must disclose on these points even when the disclosure is negative.

Sample size	no sample size calculations were performed. The sample size of data points for each assay is shown in supplementary Data 2.
Data exclusions	No data or experimental results were excluded from analysis
Replication	Each individual experiment was done with technical replicates (See figures, figure legends and supplementary data 2 for n values). Each experiment was replicated independently at least once and all reported observations were reproducible.
Randomization	Randomization was not relevant to this study as sample groups were defined by the genetic or experimental criteria of the samples.
Blinding	Blinding was not relevant to this study

Reporting for specific materials, systems and methods

Materials & experimental systems

n/a	Involved in the study	
☒	☐	Unique biological materials
☐	☒	Antibodies
☐	☒	Eukaryotic cell lines
☒	☐	Palaeontology
☐	☒	Animals and other organisms
☒	☐	Human research participants

Methods

n/a	Involved in the study	
☐	☒	ChIP-seq
☐	☒	Flow cytometry
☒	☐	MRI-based neuroimaging

Antibodies

Antibodies used	anti-Gfi1b (ARP30094_P050; Aviva Systems Biology), anti-LSD1 (ab17721;Abcam), anti-b-catenin antibody (71-2700; ThermoFisher), anti-H3K4me1 (ab8895, Abcam), anti-H3K4me2 (ab11946; Abcam), anti-H3K4me3 (ab8580; Abcam) or anti-H3K9me2 (ab1220; Abcam)
Validation	All antibodies used are commercially available and validated for the indicated uses by the manufacturers.

Eukaryotic cell lines

Policy information about cell lines

Cell line source(s)	None of the used cell lines in this study were on the list misidentified cell lines maintained by the International Cell Line Authentication Committee (ICLAC).<br>293T, K562 and U2OS cells were originally bought from ATCC.
Authentication	None of the cell lines were authenticated

Mycoplasma contamination	All cell line tested negative for mycoplasma contamination
Commonly misidentified lines (See ICLAC register)	Not applicable

Animals and other organisms

Policy information about studies involving animals; ARRIVE guidelines recommended for reporting animal research

Laboratory animals	C57BL/6 mice were maintained in a Specific-Pathogen-Free Plus environment at the Institut de recherches cliniques de Montreal (IRCM). Protocols and experimental procedures were approved by the Institutional Review Board of the IRCM and complied with IRCM and CCAC (Canadian Council of Animal Care) guidelines.
Wild animals	This study did not involve wild animals.
Field-collected samples	This study did not involve field collected samples.

ChIP-seq

Data deposition

☒ Confirm that both raw and final processed data have been deposited in a public database such as GEO.

☒ Confirm that you have deposited or provided access to graph files (e.g. BED files) for the called peaks.

Data access links
May remain private before publication.

Data generated for this study is available under GEO accession GSE117944 using reviewer access token "uvcvymsopzgvlgj".

Files in database submission

HI.4426.001.Index_8.AH_K562-H3K4me1-noWnt_R1.fastq.gz
HI.4426.001.Index_8.AH_K562-H3K4me1-noWnt_R2.fastq.gz
HI.4426.001.Index_9.AH_K562-H3K9me2-noWnt_R1.fastq.gz
HI.4426.001.Index_9.AH_K562-H3K9me2-noWnt_R2.fastq.gz
HI.4426.001.Index_10.AH_K562-H3-noWnt_R1.fastq.gz
HI.4426.001.Index_10.AH_K562-H3-noWnt_R2.fastq.gz
HI.4426.001.Index_21.AH_K562-H3K4me1-Wnt_R1.fastq.gz
HI.4426.001.Index_21.AH_K562-H3K4me1-Wnt_R2.fastq.gz
HI.4426.001.Index_22.AH_K562-H3K9me2-Wnt_R1.fastq.gz
HI.4426.001.Index_22.AH_K562-H3K9me2-Wnt_R2.fastq.gz
HI.4426.001.Index_23.AH_K562-H3-Wnt_R1.fastq.gz
HI.4426.001.Index_23.AH_K562-H3-Wnt_R2.fastq.gz
HI.4426.002.Index_8.AH_K562-H3K4me1-noWnt_R1.fastq.gz
HI.4426.002.Index_8.AH_K562-H3K4me1-noWnt_R2.fastq.gz
HI.4426.002.Index_9.AH_K562-H3K9me2-noWnt_R1.fastq.gz
HI.4426.002.Index_9.AH_K562-H3K9me2-noWnt_R2.fastq.gz
HI.4426.002.Index_10.AH_K562-H3-noWnt_R1.fastq.gz
HI.4426.002.Index_10.AH_K562-H3-noWnt_R2.fastq.gz
HI.4426.002.Index_21.AH_K562-H3K4me1-Wnt_R1.fastq.gz
HI.4426.002.Index_21.AH_K562-H3K4me1-Wnt_R2.fastq.gz
HI.4426.002.Index_22.AH_K562-H3K9me2-Wnt_R1.fastq.gz
HI.4426.002.Index_22.AH_K562-H3K9me2-Wnt_R2.fastq.gz
HI.4426.002.Index_23.AH_K562-H3-Wnt_R1.fastq.gz
HI.4426.002.Index_23.AH_K562-H3-Wnt_R2.fastq.gz
HI.4426.003.Index_8.AH_K562-H3K4me1-noWnt_R1.fastq.gz
HI.4426.003.Index_8.AH_K562-H3K4me1-noWnt_R2.fastq.gz
HI.4426.003.Index_9.AH_K562-H3K9me2-noWnt_R1.fastq.gz
HI.4426.003.Index_9.AH_K562-H3K9me2-noWnt_R2.fastq.gz
HI.4426.003.Index_10.AH_K562-H3-noWnt_R1.fastq.gz
HI.4426.003.Index_10.AH_K562-H3-noWnt_R2.fastq.gz
HI.4426.003.Index_21.AH_K562-H3K4me1-Wnt_R1.fastq.gz
HI.4426.003.Index_21.AH_K562-H3K4me1-Wnt_R2.fastq.gz
HI.4426.003.Index_22.AH_K562-H3K9me2-Wnt_R1.fastq.gz
HI.4426.003.Index_22.AH_K562-H3K9me2-Wnt_R2.fastq.gz
HI.4426.003.Index_23.AH_K562-H3-Wnt_R1.fastq.gz
HI.4426.003.Index_23.AH_K562-H3-Wnt_R2.fastq.gz
HI.4535.002.Index_1.AH_Gfi1b-negWnt3a-K652_R1.fastq.gz
HI.4535.002.Index_1.AH_Gfi1b-negWnt3a-K652_R2.fastq.gz
HI.4535.002.Index_3.AH_LSD1-negWnt3a-K652_R1.fastq.gz
HI.4535.002.Index_3.AH_LSD1-negWnt3a-K652_R2.fastq.gz
HI.4535.002.Index_8.AH_Bcat-negWnt3a-K652_R1.fastq.gz
HI.4535.002.Index_8.AH_Bcat-negWnt3a-K652_R2.fastq.gz
HI.4535.002.Index_9.AH_input-negWnt3a_R1.fastq.gz
HI.4535.002.Index_9.AH_input-negWnt3a_R2.fastq.gz
HI.4535.002.Index_10.AH_Gfi1b-posWnt3a-K652_R1.fastq.gz

HI.4535.002.Index_10.AH_Gfi1b-posWnt3a-K652_R2.fastq.gz
HI.4535.002.Index_11.AH_LSD1-posWnt3a-K652_R1.fastq.gz
HI.4535.002.Index_11.AH_LSD1-posWnt3a-K652_R2.fastq.gz
HI.4535.002.Index_22.AH_Bcat-posWnt3a-K652_R1.fastq.gz
HI.4535.002.Index_22.AH_Bcat-posWnt3a-K652_R2.fastq.gz
HI.4535.002.Index_27.AH_input-posWnt3a_R1.fastq.gz
HI.4535.002.Index_27.AH_input-posWnt3a_R2.fastq.gz
HI.4535.003.Index_1.AH_Gfi1b-negWnt3a-K652_R1.fastq.gz
HI.4535.003.Index_1.AH_Gfi1b-negWnt3a-K652_R2.fastq.gz
HI.4535.003.Index_3.AH_LSD1-negWnt3a-K652_R1.fastq.gz
HI.4535.003.Index_3.AH_LSD1-negWnt3a-K652_R2.fastq.gz
HI.4535.003.Index_8.AH_Bcat-negWnt3a-K652_R1.fastq.gz
HI.4535.003.Index_8.AH_Bcat-negWnt3a-K652_R2.fastq.gz
HI.4535.003.Index_9.AH_input-negWnt3a_R1.fastq.gz
HI.4535.003.Index_9.AH_input-negWnt3a_R2.fastq.gz
HI.4535.003.Index_10.AH_Gfi1b-posWnt3a-K652_R1.fastq.gz
HI.4535.003.Index_10.AH_Gfi1b-posWnt3a-K652_R2.fastq.gz
HI.4535.003.Index_11.AH_LSD1-posWnt3a-K652_R1.fastq.gz
HI.4535.003.Index_11.AH_LSD1-posWnt3a-K652_R2.fastq.gz
HI.4535.003.Index_22.AH_Bcat-posWnt3a-K652_R1.fastq.gz
HI.4535.003.Index_22.AH_Bcat-posWnt3a-K652_R2.fastq.gz
HI.4535.003.Index_27.AH_input-posWnt3a_R1.fastq.gz
HI.4535.003.Index_27.AH_input-posWnt3a_R2.fastq.gz
HI.4535.004.Index_1.AH_Gfi1b-negWnt3a-K652_R1.fastq.gz
HI.4535.004.Index_1.AH_Gfi1b-negWnt3a-K652_R2.fastq.gz
HI.4535.004.Index_3.AH_LSD1-negWnt3a-K652_R1.fastq.gz
HI.4535.004.Index_3.AH_LSD1-negWnt3a-K652_R2.fastq.gz
HI.4535.004.Index_8.AH_Bcat-negWnt3a-K652_R1.fastq.gz
HI.4535.004.Index_8.AH_Bcat-negWnt3a-K652_R2.fastq.gz
HI.4535.004.Index_9.AH_input-negWnt3a_R1.fastq.gz
HI.4535.004.Index_9.AH_input-negWnt3a_R2.fastq.gz
HI.4535.004.Index_10.AH_Gfi1b-posWnt3a-K652_R1.fastq.gz
HI.4535.004.Index_10.AH_Gfi1b-posWnt3a-K652_R2.fastq.gz
HI.4535.004.Index_11.AH_LSD1-posWnt3a-K652_R1.fastq.gz
HI.4535.004.Index_11.AH_LSD1-posWnt3a-K652_R2.fastq.gz
HI.4535.004.Index_22.AH_Bcat-posWnt3a-K652_R1.fastq.gz
HI.4535.004.Index_22.AH_Bcat-posWnt3a-K652_R2.fastq.gz
HI.4535.004.Index_27.AH_input-posWnt3a_R1.fastq.gz
HI.4535.004.Index_27.AH_input-posWnt3a_R2.fastq.gz
k562_h3_nownt.bw
k562_h3_wnt.bw
k562_h3k4me1_nownt.bw
k562_h3k4me1_wnt.bw
k562_h3k9me2_nownt.bw
k562_h3k9me2_wnt.bw
input_negwnt3a.bw
input_poswnt3a.bw
gfi1b_negwnt3a_k652.bw
gfi1b_poswnt3a_k652.bw
bcat_negwnt3a_k652.bw
bcat_poswnt3a_k652.bw
lsd1_negwnt3a_k652.bw
lsd1_poswnt3a_k652.bw
Gfi1b ChIP Untreated peaks.bed
Gfi1b ChIP Wnt treated peaks.bed
Bcat ChIP Untreated peaks.bed
BCat ChIP Wnt treated peaks.bed
LSD1 ChIP Untreated peaks.bed
LSD1 ChIP Wnt treated peaks.bed

Genome browser session
(e.g. UCSC)

Not applicable

Methodology

Replicates

A single replicate experiment was performed for each condition

Sequencing depth

Numbers of raw reads and aligned reads for each experiment are listed in Additional file 1

Antibodies

Samples were immuno-precipitated with 2-5 µg of either anti-Gfi1b (ARP30094_P050; Aviva Systems Biology), anti-LSD1 (ab17721;Abcam), anti-b-catenin antibody (71-2700; ThermoFisher), anti-H3K4me1 (ab8895, Abcam), anti-H3K4me2 (ab11946; Abcam), anti-H3K4me3 (ab8580; Abcam) or anti-H3K9me2 (ab1220; Abcam).

Peak calling parameters

Peaks were called using macs2 with default paramaters

Data quality	Sequencing data was verified using FastQC
Software	Reads were aligned using Bowtie2<br>Peaks were called using MACS2

Flow Cytometry

Plots

Confirm that:

☒ The axis labels state the marker and fluorochrome used (e.g. CD4-FITC).

☒ The axis scales are clearly visible. Include numbers along axes only for bottom left plot of group (a 'group' is an analysis of identical markers).

☒ All plots are contour plots with outliers or pseudocolor plots.

☒ A numerical value for number of cells or percentage (with statistics) is provided.

Methodology

Sample preparation	Bone marrow and blood samples from mice were analyzed by flow cytometry
Instrument	Hematopoietic cells were analyzed with LSR, or LSR Fortessa flow cytometer (BD Biosciences, Mountain View, CA) . For cell sorting, lineage negative BM cells were first depleted using mouse lineage cell depletion kit (Miltenyi Biotec) then applied to five-laser FACSAria II sorter (BD Biosciences).
Software	BD FACS Diva software (BD Biosciences) and FlowJo (for histogram overlays; Tree Star) were used for analysis.
Cell population abundance	An alliquote of each sorted sample was reanalysed by FACS for purity
Gating strategy	Gating strategy is preseneted in supplementary figure 1b

☒ Tick this box to confirm that a figure exemplifying the gating strategy is provided in the Supplementary Information.
