# Supplementary material for: Gfi1b regulates the level of Wnt/β-catenin signaling in hematopoietic stem cells and megakaryocytes
Source: Nat Commun. 2019 Mar 20;10:1270. doi: 10.1038/s41467-019-09273-z (PMC6426870; doi:10.1038/s41467-019-09273-z)
Supplement: Supplementary file 4 — Description of Additional Supplementary Files [file 41467_2019_9273_MOESM4_ESM.pdf]

## Description of Additional Supplementary Files

Supplementary Data 1
Expression fold change results from RNA seq

Supplementary Data 2
List of genes bound Gfi1b, LSD1 and b-catenin mouse ChIP-seq

Supplementary Data 3
List of genes bound by Gfi1b, LSD1 and b-cateninK562 ChIP-seq

Supplementary Data 4
Enhancer list bound by Gfi1b, LSD1 and b-catenin K562 ChIP-seq
